# Food Folio by Columbia Center for Eating Disorders: A Freely Available Food Image Database

**DOI:** 10.3389/fpsyg.2020.585044

**Published:** 2020-12-23

**Authors:** E. Caitlin Lloyd, Zarrar Shehzad, Janet Schebendach, Akram Bakkour, Alice M. Xue, Naomi Folasade Assaf, Rayman Jilani, B. Timothy Walsh, Joanna Steinglass, Karin Foerde

**Affiliations:** ^1^Department of Psychiatry, Columbia University Irving Medical Center, New York, NY, United States; ^2^New York State Psychiatric Institute, New York, NY, United States; ^3^Mortimer B. Zuckerman Mind Brain Behavior Institute, Columbia University, New York, NY, United States; ^4^Department of Psychology, Columbia University, New York, NY, United States; ^5^Albany Medical College, Albany, NY, United States

**Keywords:** cognition, eating, eating behavior, eating disorders, food, image, stimulus set

## Abstract

Food images are useful stimuli for the study of cognitive processes as well as eating behavior. To enhance rigor and reproducibility in task-based research, it is advantageous to have stimulus sets that are publicly available and well characterized. Food Folio by Columbia Center for Eating Disorders is a publicly available set of 138 images of Western food items. The set was developed for the study of eating disorders, particularly for use in tasks that capture eating behavior characteristic of these illnesses. It contains foods that are typically eaten, as well as those typically avoided, by individuals with eating disorders. Each image has now been rated across 17 different attributes by a large general United States population sample via Amazon’s Mechanical Turk (*n* = 1054). Ratings included subjective attributes (e.g., tastiness, healthiness, and favorable texture) as well as estimates of nutrient content (e.g., fat and carbohydrate). Each participant rated a subset of stimulus set food items (46 foods) on all 17 dimensions. Additional description of the image set is provided in terms of physical image information and accurate nutritional information. Correlations between subjective ratings were calculated and an exploratory factor analysis and exploratory cluster analysis completed. Outcomes of the factor analysis suggested foods may be described along three latent factors of healthiness, tastiness, and umami taste; the cluster analysis highlighted five distinct clusters of foods varying on these same dimensions. Descriptive outcomes indicated that the stimulus set includes a range of foods that vary along multiple dimensions and thus is likely to be useful in addressing various research questions surrounding eating behavior and cognition in healthy populations, as well as in those with eating disorders. The provision of comprehensive descriptive information allows for stimulus selection that is optimized for a given research question and promotes strong inference.

## Introduction

Food is generally considered a primary reward, and as a consequence is commonly used as a stimulus when examining processes fundamental to human behavior, including motivation, learning, and value-based decision-making (Rangel, 2013). Food images provide visual sensory input that serves as conditioned stimuli for actual food (Pavlov, 1927). Tasks involving food images have also been employed to capture eating preferences and behavior. This has not only been in attempts to explain the excess consumption of highly palatable, high-fat, foods in the context of obesity (e.g., Hendrikse et al., 2015) but also to better understand processes underlying persistent maladaptive eating behavior in eating disorders (e.g., Berner et al., 2017; Lloyd and Steinglass, 2018; Stojek et al., 2018). In this study, we introduce a new and freely available set of food stimuli, *Food Folio by Columbia Center for Eating Disorders*, and provide a deep characterization of this set, using attribute ratings collected from a large population cohort. We also consider how descriptive attributes are related and how foods group together based on the attribute ratings.

Precisely because foods possess multiple attributes (Satterthwaite and Fellows, 2018), there is potential for substantial variability between food stimulus sets used across different studies. The manner and extent of this variability is unclear, however, due to generally limited presentation of information about food stimuli in published articles (Charbonnier et al., 2016). A number of research groups have recently made food image sets publicly available, along with detailed descriptive information about the images (e.g., Satterthwaite and Fellows, 2018; Blechert et al., 2019; Toet et al., 2019). This move encourages the use of consistent stimuli across studies, which may improve the success of replication attempts. Sharing detailed image characterizations may also resolve and elucidate discrepant results through better understanding of stimulus similarities and differences between studies (e.g., van der Laan et al., 2011).

Our stimulus set, *Food Folio*, is a collection of 138 United States foods that are generally available in Western countries and may be used to address various questions surrounding cognitive processes and eating behavior. Items were specifically selected to capture and understand eating behavior in populations with eating disorders. As such, the set balances inclusion of low-fat food items that are typically eaten by those with restrictive eating disorders with the high-fat items that tend to be avoided. Eaten and avoided items were identified from previous meal study and food diary data (Mayer et al., 2012; Schebendach et al., 2012). Items were also selected to promote variation in healthiness, tastiness, and macronutrient content, and to match those featuring in our laboratory multi-item meal. The consistency of items across stimulus set and laboratory meal better enables assessment of whether tasks involving the image set predict actual eating behavior (e.g., Foerde et al., 2015, 2020). The food images have been used in tasks designed to capture the persistent selection of low-fat food items across eating disorders in the United States (e.g., Foerde et al., 2015, 2020; Gianini et al., 2019) and United Kingdom (e.g., Steinglass et al., 2015; Dalton et al., 2020). The same images have been used to study cognitive mechanisms involved in food-choice decision-making that may differentiate individuals with anorexia nervosa from healthy individuals (Bakkour et al., 2018).

In this article, we present ratings of the 138 food items in our set, along 17 different dimensions, collected in an online task from a large United States sample (*n* = 1054). Ratings include estimated macronutrient content as well as more subjective factors (e.g., taste, health, and disgust). Choice preferences in a binary food choice task are also presented. Actual nutrient content is presented alongside participant estimates, facilitating their direct comparison. Physical image properties (pertaining to color, contrast, intensity, spatial frequency, and complexity information) are described. These factors influence neural responses and are associated with subjective ratings, as well as objective nutritional information, of depicted food items (Blechert et al., 2014; Toet et al., 2019). The provision of physical image information may thus be useful for grouping foods in meaningful ways and in particular ways that serve to enable finer experimental control.

Foods varying in one dimension may vary in multiple other ways that influence participant behavior (Scheibehenne et al., 2007; Satterthwaite and Fellows, 2018). We explored this possibility within our stimulus set, by assessing correlations between different subjective rating attributes, thus informing the design and interpretation of future research using the image set. To further understand how foods are conceptualized, we explored the broad latent factors underlying variation along the 17 attribute ratings, and the clustering of foods based on these factors.

To summarize, in this article we introduce and characterize an open science resource, developed specifically for the study of eating disorders. The image set has proven useful in the study of eating disorders, and eating behavior and cognition more broadly. Use of the image set may promote consistency in methods, and replication of findings, across different research groups. Accompanying item-level and set-level descriptive information derived from a normative population can inform study design in a manner that promotes the generation of valid conclusions. This information also provides a necessary grounding for future research seeking to probe abnormal perceptions and attitudes toward food.

## Materials and Methods

### Participants

To recruit a group of individuals broadly representative of the United States population, a convenience sampling strategy was implemented using the online platform Amazon Mechanical Turk. Participants were paid $7.25 for completion of the study, which was intended to last less than 1 hour (for fair compensation). Participation was open to those aged 18 years or older and resident in the United States; there were no other exclusion criteria. A total of 1,137 individuals participated.

Before starting the study procedures, participants viewed a consent form that described procedures, measures for ensuring confidentiality, and compensation for participation. Participants provided informed consent by clicking an icon to continue with the experiment. The study was approved by the Columbia University Institutional Review Board.

### Procedure

Before commencing the food stimulus ratings, participants rated how hungry they were on a visual analog scale. Next, stimulus ratings were completed, followed by a choice task. Hunger was re-assessed after task completion and before collection of demographic information. Upon completing the study, participants were directed toward a debriefing page. This page fully detailed the study aims of understanding normative ratings of food, estimates of nutrient content, and food choice. The median time taken to complete rating and choice phases of the online experiment was 33.6 minutes.

### Food Stimuli

The stimulus set contains 138 high-resolution color photographs of food items. Each item was photographed situated on the center of a white plate, surrounded by a black background. Foods were prepared and photographed in-house by a professional photographer. The photographs used in the current task were 400 pixels in width and length, with a resolution of 72 pixels per inch.

Food items were selected to reflect a broad range of items in a standard Western diet, and included a mix of processed (e.g., pizza), unprocessed (e.g., cucumber sticks), sweet (e.g., brownie), savory (e.g., chicken fingers), single item (e.g., boiled eggs), item combinations (e.g., cereal with milk), snack foods (e.g., yogurt pretzels), and meals (e.g., rigatoni and sauce). A complete list of stimulus-set foods is included in Supplementary Table 3.

The macronutrient (grams), energy (kilocalorie) content per 100 g, and macronutrient distribution (percentage of total kilocalories) of each food were characterized using the nutrient analysis software Nutrition Data System for Research version 2009 [Nutrition Coordinating Center (NCC), 2009]. Nutritional information is available in Supplementary Table 4. The stimulus set was designed to include a balance of high-fat and low-fat food; items were classified as low-fat if <30% of total calories were from fat, and high-fat otherwise. Mean calorie and macronutrient information for low-fat and high-fat foods is in Supplementary Table 2; plots showing variability in these characteristics across food items are presented in Supplementary Figures 1, 2.

Physical image characteristics of the stimulus set were determined using Matlab scripts downloaded from the Food-Pics website^1^. Derived variables are described in full in the original study report (Blechert et al., 2014). Briefly, color variables correspond to the proportion of red, green, and blue channels averaged across all image pixels. Size reflects the proportion of non-white pixels relative to white pixels in the image. Intensity is the difference in mean luminance of the non-white pixels relative to white pixels, and normalized intensity is intensity adjusted for object size. Within-object contrast is the SD of luminance across non-white pixels. Spatial frequency content was calculated, allowing quantification of median power, defined as the variation in pixel luminance across different spatial scales. Image complexity is the number of pixels representing contour outlines, while normalized complexity is the proportion of image pixels representing contour outlines. Values for these measures (at the summary and individual food level) are provided in Supplementary Tables 1, 5.

The food stimulus set and accompanying descriptive data for each image are available to download here: https://osf.io/483mx/.

### Experimental Task

Each participant was allocated a stimulus set that contained 46 of the total 138 foods, to keep testing session duration at approximately 1 hour. Stimulus sets were created by dividing food stimuli into six subgroups (of 23 items), each including a similar selection and variety of foods, and assigning two subgroups to each stimulus set list. Completing all possible pairings of subgroups resulted in 15 stimulus sets, which were randomly assigned to participants. High-fat foods constituted 44% or 49% of all foods in each list. This strategy ensured participants rated a variety of food items and that the ratio of low- to high-fat food items rated was fairly constant across participants. At the same time, there was a level of consistency in the food items that appeared together across participants, which better allowed for comparison of attribute ratings, and exploration of associations between attribute ratings, within the stimulus set. Each food was rated by between 328 and 375 participants (precise information is available in Supplementary Table 3).

### Stimulus Rating

Participants rated each item along 17 separate attributes: protein content, fat content, carbohydrate content, vitamin content, sodium content, sugar content, gluten content, caloric content, healthiness, tastiness, how tasty others think the food is, fillingness, savoriness, pleasantness of texture, disgustingness, feelings resulting from the food (happiness or sadness), and familiarity. Each participant provided a total of 782 ratings (17 ratings for each food in their 46-item set).

**FIGURE 1:**
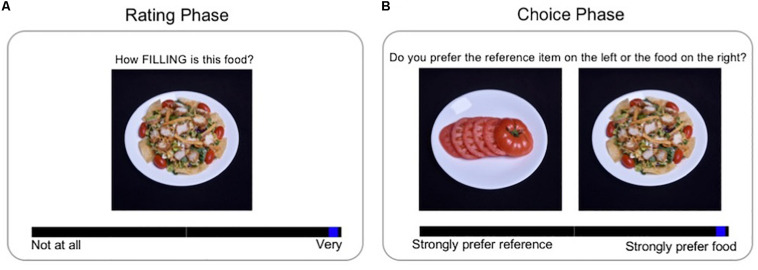
Food rating and choice tasks. The task consisted of two phases: Rating and Food Choice. In the Rating phase **(A)**, participants viewed each food in their 46-item set consecutively and provided ratings for a particular attribute; this was repeated for each of the 17 attributes. On each trial in the Choice phase **(B)**, participants indicated their preference for each of the non-reference food items in their set relative to the repeated reference item (previously rated neutral on health and taste).

The Instructions screen told participants that they would be viewing a series of foods and asked to provide ratings based on only their opinions, and that there were no right or wrong answers. They were asked to think about a snack-sized portion of the food rather than the exact amount of food shown in the picture when rating the foods. For each attribute, participants rated each of the food items in their 46-item set consecutively, using a slider scale displayed below the food item. The slider scale ranged from low to high endorsement (e.g., low tastiness to high tastiness; Figure 1). Participants could also give neutral ratings by positioning the slider in the middle of the scale. Although numeric values were not visible, the slider position corresponded to a continuous numerical value, with two decimal places, ranging from 0 (low endorsement) to 10 (high endorsement). The left/right placement of rating scale anchors for each attribute (e.g., rating content from low to high vs. high to low) was counterbalanced across participants, but was kept consistent within each participant across rating tasks. For the precise wording of the rating questions, and corresponding scale anchors, see Table 1. The order of rating each of the 17 attributes was randomized for each participant, and each food item was presented in a random order within each attribute rating.

**TABLE 1:** Rating task questions and response anchors.

**Question**	**Response range**
**How high is the food in CALORIES?**	Low–high
**How high is the food in CARBOHYDRATES?**	Low–high
**How high is the food in FAT?**	Low–high
**How high is the food in GLUTEN?**	Low–high
**How high is the food in VITAMINS?**	Low–high
**How high is the food in SUGAR?**	Low–high
**How high is the food in PROTEIN?**	Low–high
**How high is the food in SODIUM?**	Low–high
**How does this food make you FEEL?**	Very sad–very happy
**Generally, how TASTY would other people think this food is?**	Not at all–very
**How TASTY is this food?**	Good–bad
**Is this food more SWEET or SALTY/SAVORY?**	Sweet–salty/savory
**How HEALTHY is this food?**	Unhealthy–healthy
**Do you like the TEXTURE of this food?**	Not at all–very much
**How DISGUSTING is this food?**	Not at all–very
**How FILLING is this food?**	Not at all–very
Do you know what this type of food is?*	Don’t know–have eaten before

*This indexes familiarity and had a midpoint anchor of “know but have never eaten.”

### Choice Task

After all ratings had been provided, participants were instructed to choose what they would prefer to eat “right now,” between a reference food and each of the other 45 foods in their specific stimulus set, across 45 consecutive trials. The reference food was a randomly selected item from those the participant had rated neutrally on both tastiness and healthiness. A neutral rating was one in the middle of the participants own rating range; for example, if participants rated all foods as between 4 and 8 for tastiness, a neutral tastiness rating would correspond to a value of 6.

The reference food appeared on the same side of the screen on each Choice trial (i.e., on the left or right). The alternative food appeared on the other side of the screen and was updated in each new trial. Participants were asked to imagine eating a snack-sized portion of each presented food while making their choices. Preferences were indicated using a slider scale that appeared below the images, and that was anchored by a strong preference for the reference food (corresponding to the numerical value of 0), to a strong preference for the alternative food (10) (Figure 1). The midpoint on the slider scale corresponded to no preference. As in the Rating phase, the numeric values associated with slider position were not visible to participants, and left/right placement of reference/alternate food was counterbalanced across participants.

Before both the Choice and Rating phases, participants completed practice trials. Data from the practice trials were not analyzed.

### Hunger Rating

Hunger ratings were provided using a visual analog scale that ranged from “Not at all” (0) to “Extremely” (10).

### Data Analysis

Analyses of the rating and choice data were completed using R (R Core Team, 2017). Data from 43 individuals whose self-reported values for height and weight were uninterpretable (e.g., resulting in a negative BMI) were excluded. Individuals with a BMI below the minimum healthy level of 18.5 kg/m^2^ (*n* = 31) and above 60 kg/m^2^ (*n* = 9) were also excluded from the current analyses. This was to promote the generalizability of results, given the low estimated prevalence of BMIs outside of the 18.5–60 kg/m^2^ range (Fryar et al., 2018; Hales et al., 2018). The resulting sample size for the current study was 1,054. Removing the small number of participants (52) who met criteria for problematic eating attitudes or behavior [scoring 20 or higher on the Eating Attitudes Test-26; EAT-26 (Garner et al., 1982)] did not affect the results. Given this, and the fact that the EAT-26 does not constitute a formal diagnosis of eating disorder, these individuals were retained in analyses. In total, 11.3% of participants reported a food allergy; these participants were also retained given allergies can be expected to impact a minimal number of trials for each individual, particularly as each participant was presented with only a subset of food items.

Mean ratings for each of the 17 attributes, for each of the 138 foods, were calculated, using participants’ raw scores. Mean values for attribute ratings were calculated across all foods, and for low-fat and high-fat foods separately. The continuous choice variable, the outcome of the Food Choice Task, was converted into a binary variable that indicated preference for the non-reference food or the reference food. The proportion of times each food was selected over the reference item, across all participants, was calculated. Item-level choice data, as well as averages for low-fat and high-fat categories, and across all foods of the set, are presented in Supplementary Tables 2, 3, as these were not primary outcomes of the current study.

Pearson correlation coefficients were calculated to index the pairwise association between each of the 17 subjective rating attributes, using the mean attribute ratings for each food item (across all participants). In addition, Pearson correlation coefficients were calculated to measure the relationship between average participant ratings of macronutrient/calorie content and actual macronutrient (both in grams and as percentage of total calories)/actual calorie content.

Exploratory factor analysis (EFA) was completed using the mean attribute scores for each food (i.e., 17 different ratings for each of the 138 foods). To ease factor interpretation, before deriving average attribute ratings to input into the factor analysis, scores corresponding to participant estimates of calorie, fat, carbohydrate, gluten, sodium, and sugar content were reversed so that higher ratings reflected lower estimated content. This was achieved by deducting the given rating from the maximum possible rating of 10.00. The EFA used a maximum likelihood estimation method, implemented by the factanal function of the psych package (Revelle, 2019). A varimax rotation method was used to minimize cross-factor loadings. The number of factors specified in the fitting procedure was based on outcomes of the Cattell–Nelson–Gorsuch (CNG) scree test (Gorsuch and Nelson, 1981), completed using the nCng function of the nFactors package (Raiche, 2010). The factors that emerged as underlying the 17 attribute ratings were characterized by examining the loadings of the attribute ratings on the factors; 0.50 was the threshold for considering an attribute to load on a given factor, as the distribution of attribute ratings varied (Comrey and Lee, 1992).

Standardized factor scores for each food in each participant’s rating set were calculated, using Thomson’s regression method (Thomson, 1951); rating attribute coefficients used in the regression equation were determined from factor loadings and the rating dimension covariance matrix. Average factor scores per food, and for foods in low-fat and high-fat categories, were then calculated using all participant ratings.

Taking average factor scores for each food, the foods were clustered using the k-means algorithm from the stats package (R Core Team, 2017). To determine the optimal number of clusters, scree plot, silhouette, and gap statistic methods were used, along with qualitative analysis of resulting cluster characteristics (i.e., mean factor scores), and cluster contents. Mean values for each latent factor, and rating attribute, were calculated for each cluster of foods, using all participant ratings. Mean nutrient content values (i.e., objective nutritional information) for foods in each cluster were also calculated.

Given the descriptive intentions of the current exploratory analyses, SDs are reported along with mean values. Adjustment for the non-independence of data points was not required.

## Results

### Demographics

**FIGURE 2:**
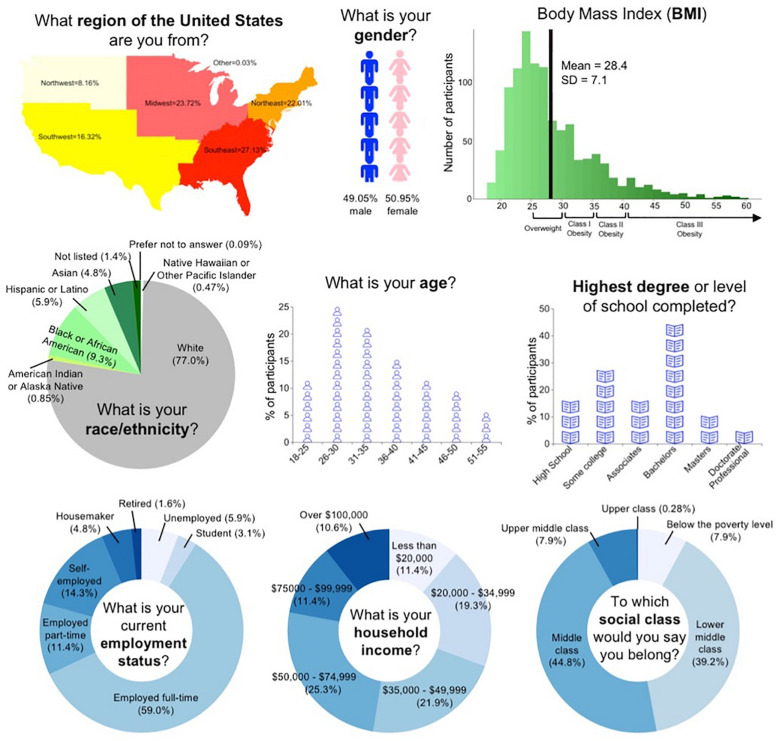
Participant demographic characteristics.

Participant demographics are presented in Figure 2 and Supplementary Table 1. Approximately half of the participants were female. Though the majority were white and middle-class, participants were recruited from across the United States, and showed diversity in age, BMI, income, and educational attainment.

### Hunger

The mean hunger rating was 4.80 (SD = 2.56) before task completion and 5.81 (SD = 2.80) post-task completion – a significant change in hunger over time [repeated measures *t*-test: *t*(1053) = 16.28, *p* < 0.001].

### Mean Ratings of Food Images

**FIGURE 3:**
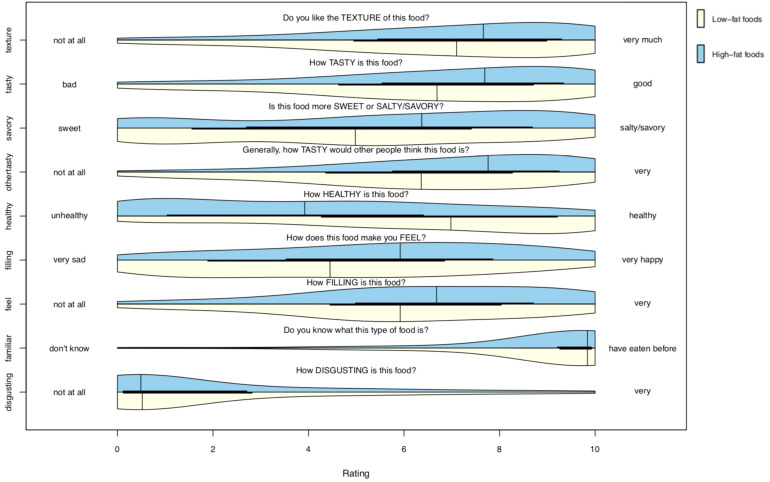
Probability distribution plot of food characteristic ratings. Plots show the distribution of scores for each food characteristic rating across the collection of low-fat (lower/yellow) and high-fat (upper/blue) foods of the stimulus set. Every rating (for each of the 46 foods, from all 1,054 participants) is included. N ratings for low-fat foods = 25,647; n ratings for high-fat foods = 22,862.

**FIGURE 4:**
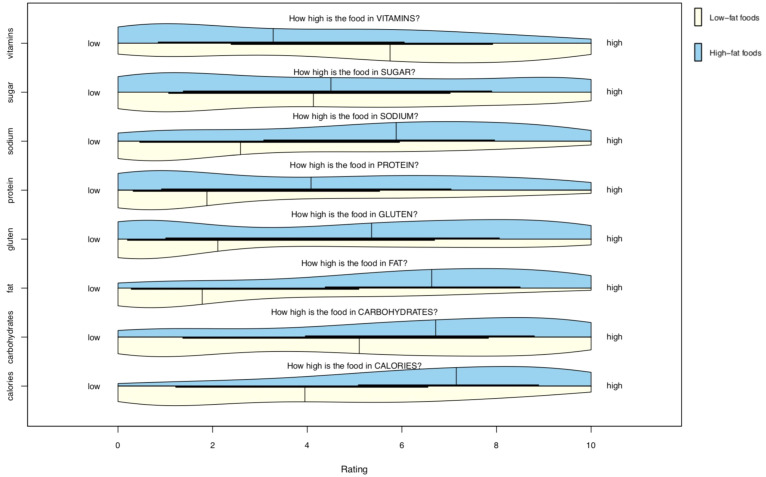
Probability distribution plot of estimated nutritional content ratings. Plots show the distribution of scores for each rating of nutritional content across the collection of low-fat (lower/yellow) and high-fat (upper/blue) foods of the stimulus set. Every rating (for each of the 46 foods, from all 1,054 participants) is included. N ratings for low-fat foods = 25,647; n ratings for high-fat foods = 22,862.

The distributions of mean attribute ratings for low-fat and high-fat foods are shown in Figure 3 (food characteristics) and Figure 4 (nutritional content). Summary-level information for the stimulus set is included in Supplementary Table 2 and item (per food)-level data in Supplementary Table 3.

High-fat foods had greater scores across 13 of the 17 subjective rating dimensions. High-fat foods had lower scores on healthiness and estimated vitamin content, and were similar to low-fat foods in terms of disgust and familiarity ratings.

### Correlations Among Attributes

**FIGURE 5:**
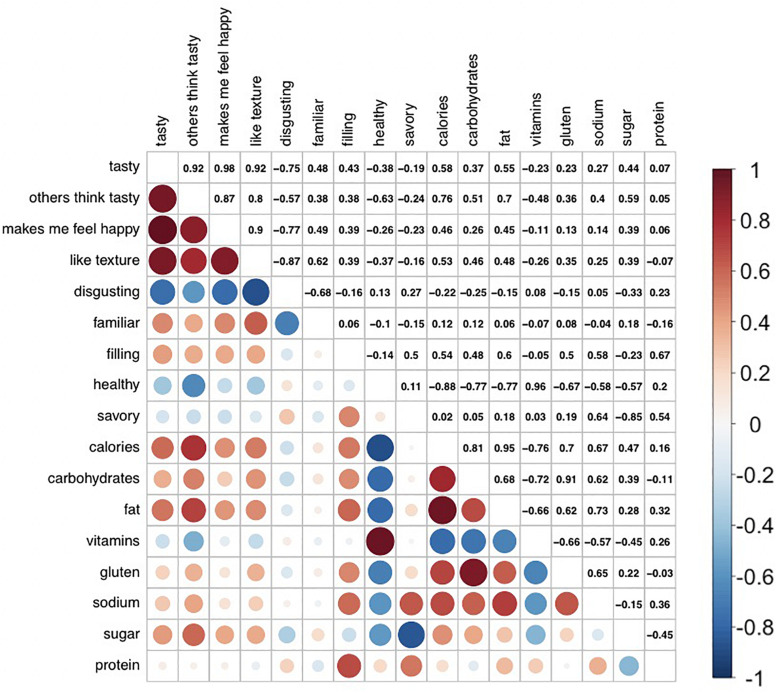
Correlations between subjective rating dimensions across stimulus set. The matrix shows Pearson R correlations (described using a heat-map function and numeric values), which index the strength of association between each pair of attribute ratings. The mean for each attribute rating, for each food, was calculated using all available participant ratings. Correlations between average attribute ratings across all foods were then determined, indicating strong correlations between multiple of the attributes.

The correlation analysis using the mean values of the subjective attribute ratings for each food demonstrated strong correlations between multiple attributes (Figure 5).

**FIGURE 6:**
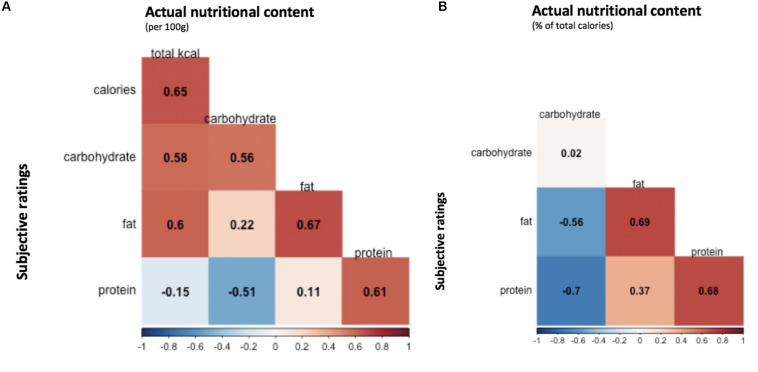
Average correlations between subjective ratings and actual nutritional content. Correlations between average participant ratings for calorie, carbohydrate, fat and protein content, and actual nutrient content values were estimated across the 138 food items. **(A)** Pearson correlation coefficients reflecting the association between participant ratings of nutrient content and actual calorie/macronutrient content per 100 g. **(B)** Pearson correlation coefficients reflecting the association between participant ratings of nutrient content and actual macronutrient content as a percentage of total calories.

Correlations between average subjective ratings of macronutrient/calorie content and actual nutritional content are shown in Figure 6.

### Factor Analysis

**FIGURE 7:**
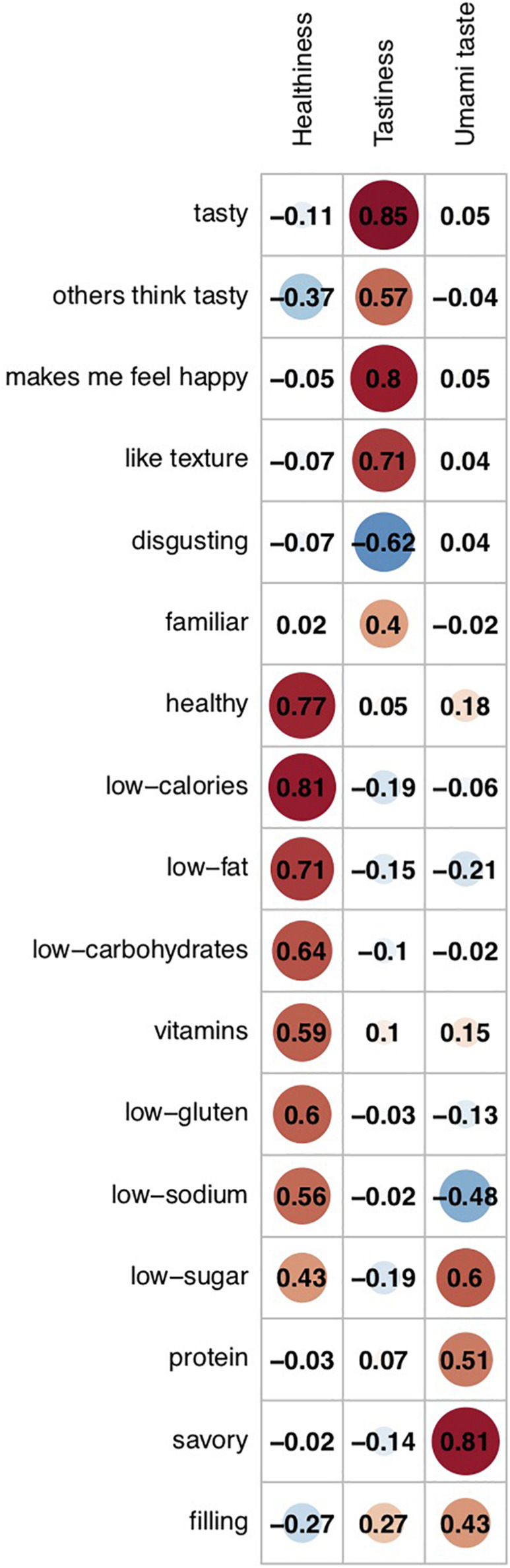
Loadings of rating attributes on the three latent factors. Ratings for calories, fat, carbohydrates, gluten, sodium, and sugar were reversed so that higher ratings indicated lower content of these nutrients. The corresponding attributes were relabeled accordingly (e.g., calories became low calories). Factor 1 was labeled as Healthiness given positive loadings from low-calorie, low-carbohydrate, low-fat, low-gluten, and low-sodium content ratings, and positive loadings from healthiness and vitamin content ratings. Factor 2 was labeled Tastiness based on positive loadings from ratings of tastiness, estimates of others’ tastiness evaluations, favorable texture, feelings of happiness evoked from the food, and negative loadings from disgust ratings. Factor 3 was labeled as an umami taste factor, given the positive loading of savoriness ratings, and (to a lesser extent) ratings of protein and low-sugar content.

Using the correlation matrix output (see Figure 5), the CNG test indicated that a three-factor solution best explained variation along the 17 rating dimensions. The exploratory factor analysis confirmed that the three-factor solution explained 49.3% of the total variance in rating scores, with factor 1 explaining 21.1% variance, factor 2 explaining 17.3% variance, and factor 3 explaining 10.8% variance. The loading of each of the attributes on the three factors is presented in Figure 7. Factor 1 was labeled as a Healthiness factor, based on positive loadings from the low-calorie, low-carbohydrate, low-fat, low-gluten, and low-sodium content attributes, and positive loadings from healthiness and vitamin content ratings. Factor 2 was labeled as Tastiness, based on positive loadings from ratings of tastiness, estimates of others’ taste evaluations, favorable texture, feelings of happiness evoked from the food, and negative loadings from disgust ratings. Factor 3 was labeled as an umami taste factor, given the positive loading of savoriness ratings, with savory being considered the umami taste (Ikeda, 1909), and (to a lesser extent) ratings of protein and low-sugar content.

The average factor scores for each of the 138 foods are presented in Supplementary Table 6. Summaries across all foods, and by low- and high-fat category, calculated using all food-item ratings from all participants, are detailed in Supplementary Table 7. On average, high-fat foods scored positively on the factors of tastiness and umami taste, and negatively on the factor of healthiness; the opposite was true for low-fat foods.

**FIGURE 8:**
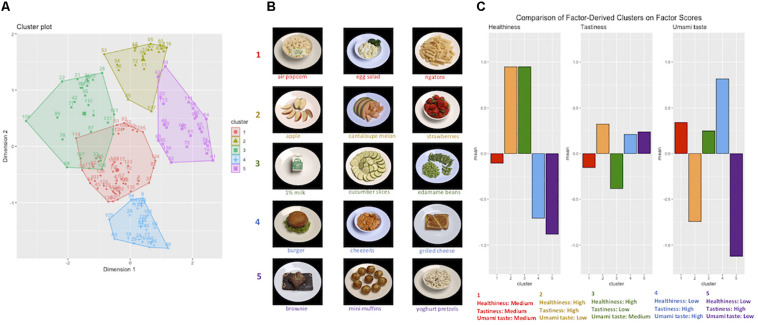
Cluster analysis of stimulus set foods based on latent factor scores. Three latent factor scores (reflecting healthiness, tastiness, and umami taste) were calculated for each food item in each participant’s set, using Thomson’s regression method and ratings for each of the 17 rated attributes. Average factor scores for each food could then be calculated across participants. **(A)** Foods were clustered into five categories based on their average factor scores. **(B)** Examples of food items within each cluster. **(C)** Mean factor scores for each cluster of foods were calculated, using all participant data (i.e., 46 data points for each factor, for each of the 1,054 participants), demonstrating the five clusters varied on the latent dimensions of healthiness, tastiness, and umami taste.

Results of the clustering analysis suggested that foods could be meaningfully clustered into five groups based on average factor scores for each food. Outcomes of application of the k-means clustering algorithm with five clusters specified are displayed in Figure 8. Details of the items in each cluster are given in Supplementary Table 8.

Analysis of average factor scores for foods in each cluster, using the full set of participant ratings, supported between-cluster variability on the three factors (see Table 2 and Figure 8). Average scores across foods in each cluster, with respect to objective characteristics and attribute ratings, are detailed in Supplementary Table 9 and Supplementary Figures 2, 3.

**TABLE 2:** Mean factor scores for foods in each cluster.

**Factor**	**Cluster 1 (*n* = 15,745)**		**Cluster 2 (*n* = 6,729)**		**Cluster 3 (*n* = 9,161)**		**Cluster 4 (*n* = 8,438)**		**Cluster 5 (*n* = 8,436)**	
**Healthiness**	−0.11	0.59	0.95	0.59	0.95	0.68	−0.71	0.60	−0.88	0.56
**Tastiness**	−0.15	0.88	0.32	0.89	−0.38	1.05	0.21	0.81	0.24	0.85
**Umami taste**	0.34	0.65	−0.74	0.57	0.25	0.56	0.82	0.51	−1.12	0.63

*n, number of ratings.*

Studying cluster characteristics in terms of the average factor scores suggested the clusters could be summarized as follows: cluster 1 – moderate for all latent factors; cluster 2 – healthy, tasty, low-umami; cluster 3 – healthy, not tasty; cluster 4 – unhealthy, tasty, high-umami; cluster 5 – unhealthy, tasty, low-umami.

### Sensitivity Analyses

Analyses were rerun with the (76-item) subset of foods that has been used as part of the Food Choice Task in multiple existing studies with eating disorder populations (e.g., Foerde et al., 2015; Dalton et al., 2020). Differences between low-fat and high-fat foods were more pronounced in analyses with the 76-item subset; however, outcomes did not qualitatively differ from those of the primary analysis, and are presented in the Supplementary Information (Part 2) and Supplementary Tables (File 2).

The main analyses were also rerun excluding the 2,980 trials (of 48,509) on which the food item was rated as less than 5 for familiarity, indicating that the food was not well recognized by the participant. Outcomes did not qualitatively differ from those of the main analyses, except for average familiarity ratings, which increased at the item and set level. Item- and summary-level rating data from these analyses are presented in Supplementary Tables 10, 11.

## Discussion

In this study, ratings from a large general population sample were used to carefully characterize *Food Folio*, a free-to-download image database comprising 138 food items. Relative to other available image sets, *Food Folio* has been characterized in greater detail, including across ratings along 17 different dimensions, as well as actual nutrient content and physical image properties (Blechert et al., 2019). The image set and normative data are publicly available to encourage consistency across studies using food images and promote replication of research findings.

Although images were compiled with the intention of studying disordered eating, the characterization presented here demonstrates that the *Food Folio* stimulus set may be used to address a variety of research questions relating to normative eating behavior and cognition. Foods were highly familiar, generally liked, and showed variation along most of the 17 rated attributes, suggesting utility of the stimulus set in probing the influence of various attributes on value computations and eating behavior. The complexity of food stimuli in this set supports their use in tasks designed to elucidate processes and mechanisms underlying evaluation of multi-attribute stimuli (e.g., Suzuki et al., 2017), and individual differences in these processes (e.g., Sullivan et al., 2015).

The detailed descriptive information that accompanies our images may be used to guide the creation of stimulus subsets optimized for addressing specific research questions. For example, it may be of interest to further understand how information surrounding different nutrients is integrated to form a broader and more abstract construct such as healthiness. In this case, a set of foods varying meaningfully in estimated sugar, fat, and salt content might be required. Such a set may be identified from our database, using the available rating information.

The descriptive information may also promote reliability of findings and conclusion validity. In one scenario, researchers may wish to measure the influence of expected fillingness of a food on choice preference. The correlation analyses indicated strong associations between multiple rated attributes. For example, fillingness was highly correlated with estimates of protein, sodium, and fat content. A consequence of this is that behavior assumed to be in response to one characteristic (e.g., fillingness) could viably be explained by alternative factors (e.g., estimated nutrient content). The stimulus information presented allows for the development of a set of foods that are decorrelated along the dimension of interest and nuisance factors, promoting straightforward inference and the replication of results.

Participant estimates of nutritional content were generally well correlated with the values derived from nutritional analysis software, indicative of good nutrition knowledge in the general population. This suggests that research participants evaluate food stimuli as intended by researchers, supporting the validity of conclusions drawn from studies using these stimuli. Conversely, if participants were inaccurate in their assessment of food macronutrient content, conclusions surrounding the influence of macronutrients on choice could be misleading.

The data also confirmed the expected distinctions between images of high-fat and low-fat foods, with high-fat foods rated as higher in calorie content, tastier, and less healthy compared with low-fat foods. Low-fat and high-fat foods were on average well matched for attributes on which they would not necessarily be expected to differ, such as disgust and familiarity, and which affect consumption and are associated with particular patterns of neuronal activation (Fallon and Rozin, 1983; Steptoe et al., 1995; Weierich et al., 2010; Pujol et al., 2018). This supports the fairly straightforward interpretation of differential responses to low-fat and high-fat foods (i.e., differences are not explained by factors not considered by researchers) when using Food Folio. Likely due to the consistent presentation of foods in our image set (foods situated on a white plate against a black background), across the entire stimulus set there was very little variation in physical image properties that affect neurocognitive responses. This information is relevant for neuroimaging studies using Food Folio, and suggests inter-image differences in physical characteristics will introduce minimal risk of bias when probing neural correlates of certain processes.

The factor analysis indicated three separable factors could explain variation along the correlated rating dimensions: tastiness; healthiness; and umami taste. Thus, results from this large, general population study are consistent with the assumption that tastiness and healthiness comprise independent value systems, which is reassuring given that this assumption has been central to a series of food choice tasks aimed at understanding dietary restriction (e.g., Hare et al., 2009, 2011; Foerde et al., 2015; Maier et al., 2015). These studies have presented foods varying in healthiness and tastiness to determine their relative influence on choice, elucidating the mechanisms underlying selection of healthier items over those considered tastier, which has implications for weight and health.

The umami taste factor largely indexed savoriness, and its emergence as one of only three latent factors underlying the 17 attribute ratings suggests it is a particularly salient dimension of foods. This supports the importance of incorporating variation in savoriness into choice paradigms aimed at capturing food preferences (Oustric et al., 2020). The observed association between savoriness ratings and estimated protein content is consistent with previous findings (van Dongen et al., 2012; Lease et al., 2016), and with the suggestion that ability to detect savory or umami taste serves as a means of detecting the protein content of food (e.g., Luscombe-Marsh et al., 2008; Griffioen-Roose et al., 2012).

The outcomes of the cluster analysis indicated that the set of 138 foods formed five distinct clusters based on the mean healthiness, tastiness, and umami taste factor scores (calculated across participants). The five clusters differed meaningfully along the latent dimensions of tastiness, healthiness, and umami taste. Previous neuroimaging findings suggest distinct neural signatures of foods in categories similar to those emerging from our cluster analysis (King et al., 2018), supporting the validity of organizing food items based on the cluster analysis output.

The division of foods according to their umami taste further supports the salience of this characteristic, aligning with the existence of distinct taste pathways for sweet and umami tastes in the mouth, gut, and central nervous system (Rolls, 2009; Lee and Owyang, 2017; Roper and Chaudhari, 2017). The clear separation between clusters containing healthy and unhealthy foods, and the presence of a more neutral category containing sources of starchy carbohydrates and protein, suggests that foods are conceptualized in a manner consistent with published guidance on food groups (Herforth et al., 2019). This supports the influence of public health information on how we think about food, which has implications for the success of population-level behavior change initiatives. Consideration of the way in which multi-dimensional foods are typically organized may also inform interventions designed to promote more varied diets. Mapping foods in a two-dimensional space facilitates identification of avoided foods similar to those with which one is familiar or comfortable with eating, to perhaps encourage small and gradual changes in eating behavior that cumulatively have a large impact.

The normative data presented demonstrate how our set of food stimuli are perceived and categorized in a healthy population sample. This is necessary for the development of novel questions concerning the predictive and causal effects of divergence from typical food stimulus characterization (e.g., attribute associations, sensitivity to variation in particular sensory characteristics) on eating pathology. Addressing these questions may further our understanding of how eating disorders develop and are maintained, and has potential to improve diagnosis of these conditions.

The internet-based nature of the study increased the feasibility of participation, enabling us to collect ratings from a large population sample, which in turn enhances the likelihood that these ratings are reflective of beliefs and preferences of individuals in the general population. The trade-off for this aspect of generalizability is the inability to standardize certain aspects of participation, for example the time at which the study was completed or the time since the last meal was consumed. This introduces some variability into the data, which is likely to be inconsequential in large studies, but could affect the reliability of conclusions in smaller studies. Variability similarly results from the inclusion of trials in which participants were unfamiliar with, or allergic to, the depicted food items. Characteristics of the food items themselves may also have introduced noise: for example, calorie content was not constant across images. We note that attempting to keep calorie content constant would necessarily have introduced complications for other ratings (e.g., fillingness) because portion sizes would have been altered. We hope that providing information about the images can minimize, or mitigate effects of, sources of unwanted variability. For instance, use of the accompanying information allows for the selection of items that are familiar to a normative United States sample, or statistical adjustment for unavoidable forms of variability. Certain additional considerations should be made when interpreting the rating data. First, although the sample includes a wide range of individuals who vary across many demographic dimensions, the sample is not representative of the United States as a whole, limiting generalizability of rating data to the entire United States population. A greater proportion of the United States population is considered overweight or obese relative to our sample (71.6 vs. 62.8%; National Center for Health Statistics, 2018). Our sample also included a greater proportion of non-Hispanic white participants (77.0% relative to 60.2% in United States population; US Census Bureau, 2018a) and was more educated relative to the wider United States population; for example, 12.5% were not educated beyond high-school level, compared with 39.3% of the United States (US Census Bureau, 2018b). Second, the task was completed online with no way of ensuring that participants’ attention was maintained. That ratings generally aligned with actual nutritional content suggests that participants were sufficiently attentive to respond accurately. Third, the outcomes of the choice task were hypothetical, which may result in responses that reflect intention more than behavior (Camerer and Mobbs, 2017), although previous studies do find hypothetical food choices to be predictive of actual eating behavior (Medic et al., 2016). Finally, hunger increased during the task, which could have affected ratings. The risk of systematic bias resulting from this is likely to be low, given the relatively small increase in hunger and the fact that both food items and rating tasks were presented in a random order.

To conclude, *Food Folio* is a publicly available set of food images. Though compiled for the study of disordered eating, ratings from a large population sample support the utility of the set in addressing a variety of research questions pertaining to cognition and eating behavior within non-clinical populations. The ratings also support the validity of existing understandings of value systems and conceptualization of food items/food groups. The normative rating data and information regarding nutritional content/physical image properties that accompanies the image set should allow optimization of studies to address research questions of interest, in turn promoting the replicability and integrity of findings.

## Data Availability Statement

The original contributions presented in the study are included in the article/Supplementary Material, further inquiries can be directed to the corresponding author.

## Ethics Statement

The studies involving human participants were reviewed and approved by the Columbia University Institutional Review Board. The participants provided their written informed consent to participate in this study.

## Author Contributions

ZS, JS, and KF conceived the study. AB, AX, NA, BTW, JS, and KF contributed to study design. AX and RJ assisted with data collection. ECL and ZS completed the data analyses. ECL drafted the manuscript. All authors refined the manuscript and approved the final version for submission.

## Conflict of Interest

The authors declare that the research was conducted in the absence of any commercial or financial relationships that could be construed as a potential conflict of interest.

## Footnotes

1 http://food-pics.sbg.ac.at
