# Supplementary material for: Food Folio by Columbia Center for Eating Disorders: A Freely Available Food Image Database
Source: Front Psychol. 2020 Dec 23;11:585044. doi: 10.3389/fpsyg.2020.585044 (PMC7785939; doi:10.3389/fpsyg.2020.585044)
Supplement: Supplementary file 3 [file Data_Sheet_1.PDF]

Supplementary Information for

Food Folio by Columbia Center for Eating Disorders: A freely available food image database

Lloyd et al.

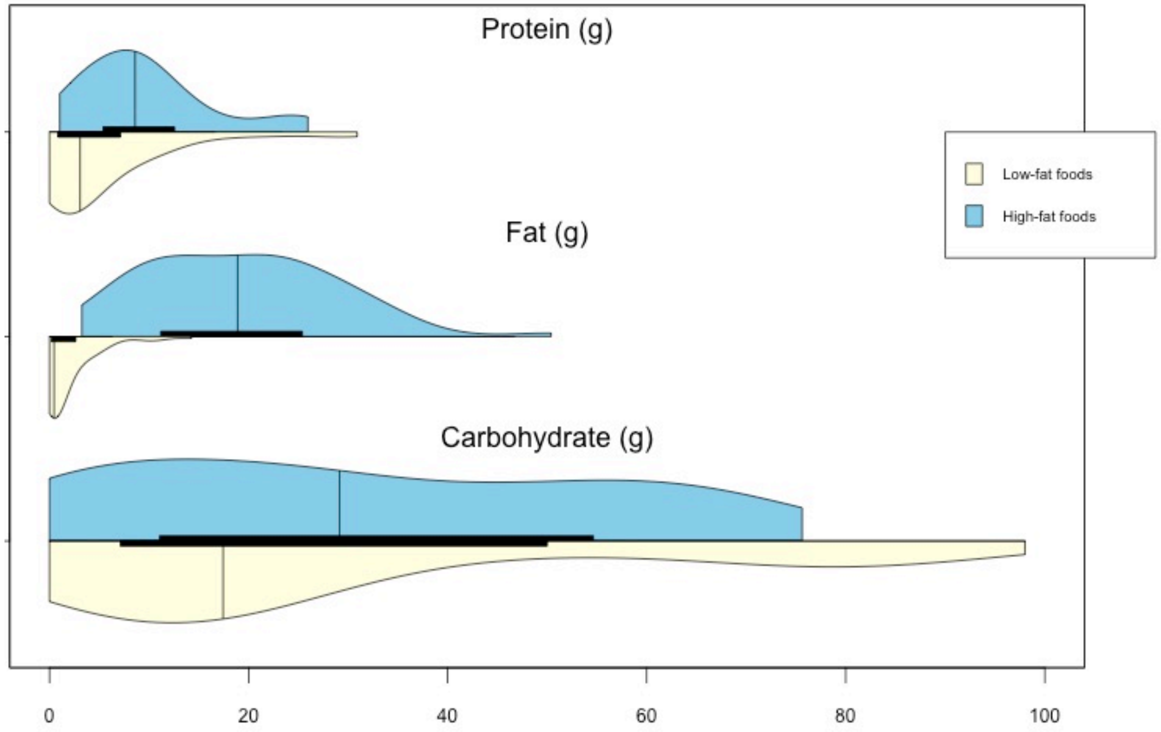

Figure 1: Probability distribution plot showing nutrient content across items of the stimulus set (grams)

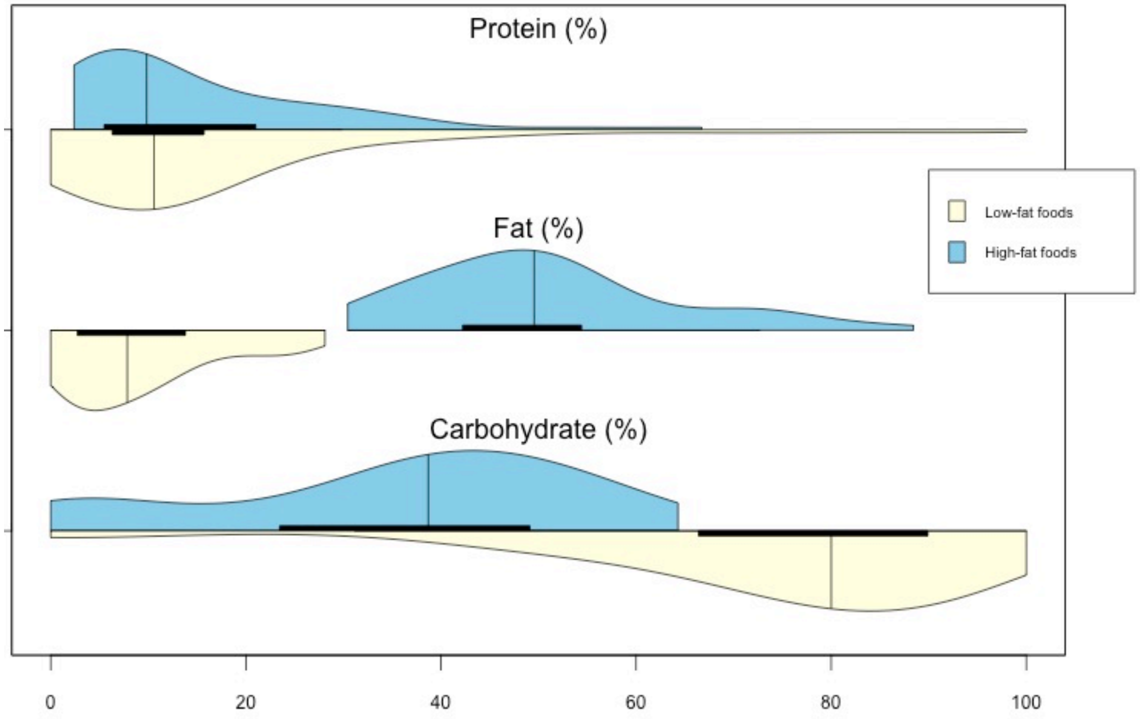

Figure 2: Probability distribution plot showing nutrient content across items of the stimulus set (as % of total calories)

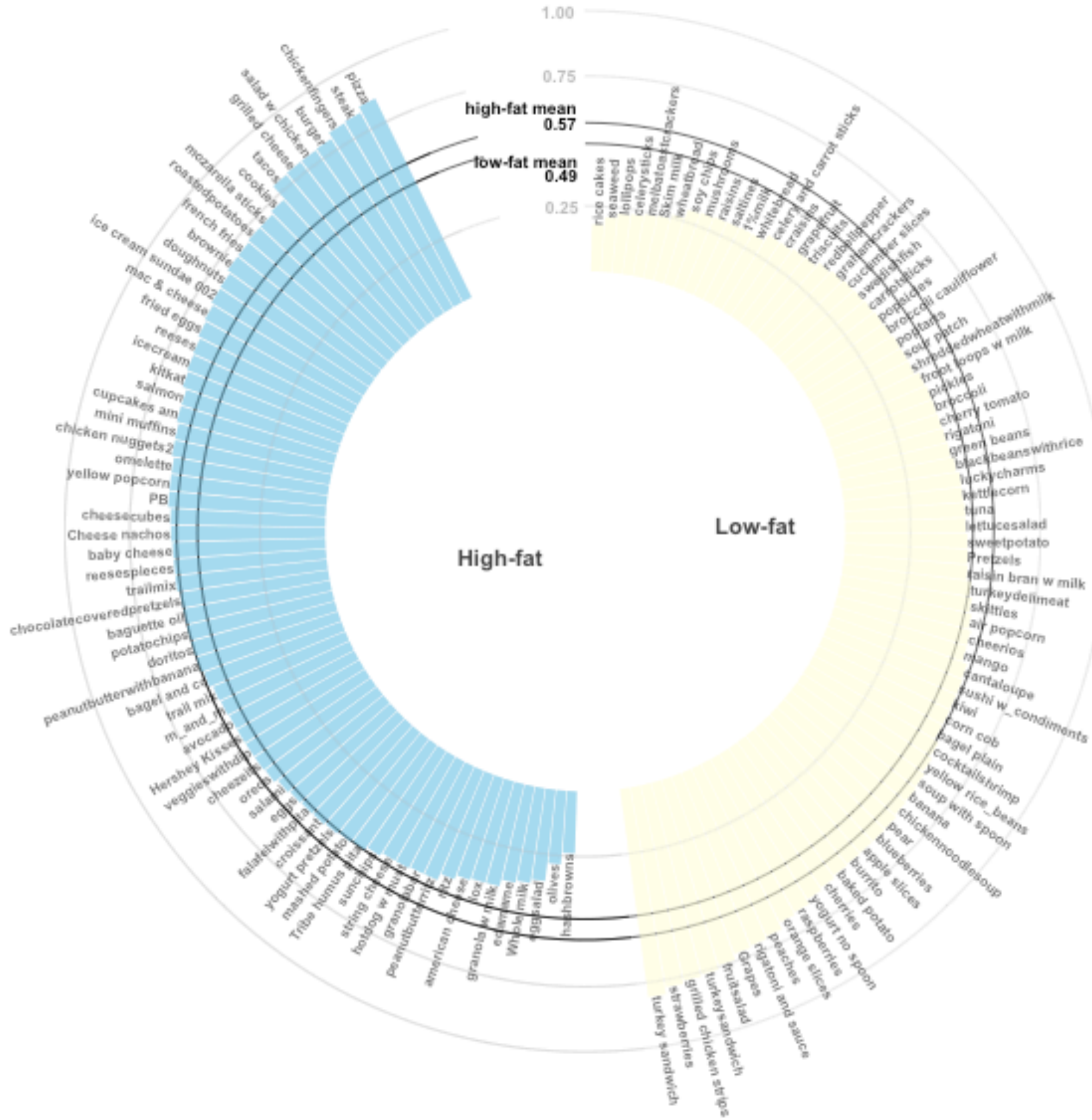

Figure 3: Selection of Each Food in Choice Phase

The proportion of times each food was selected over the reference item in the choice phase was calculated across participants

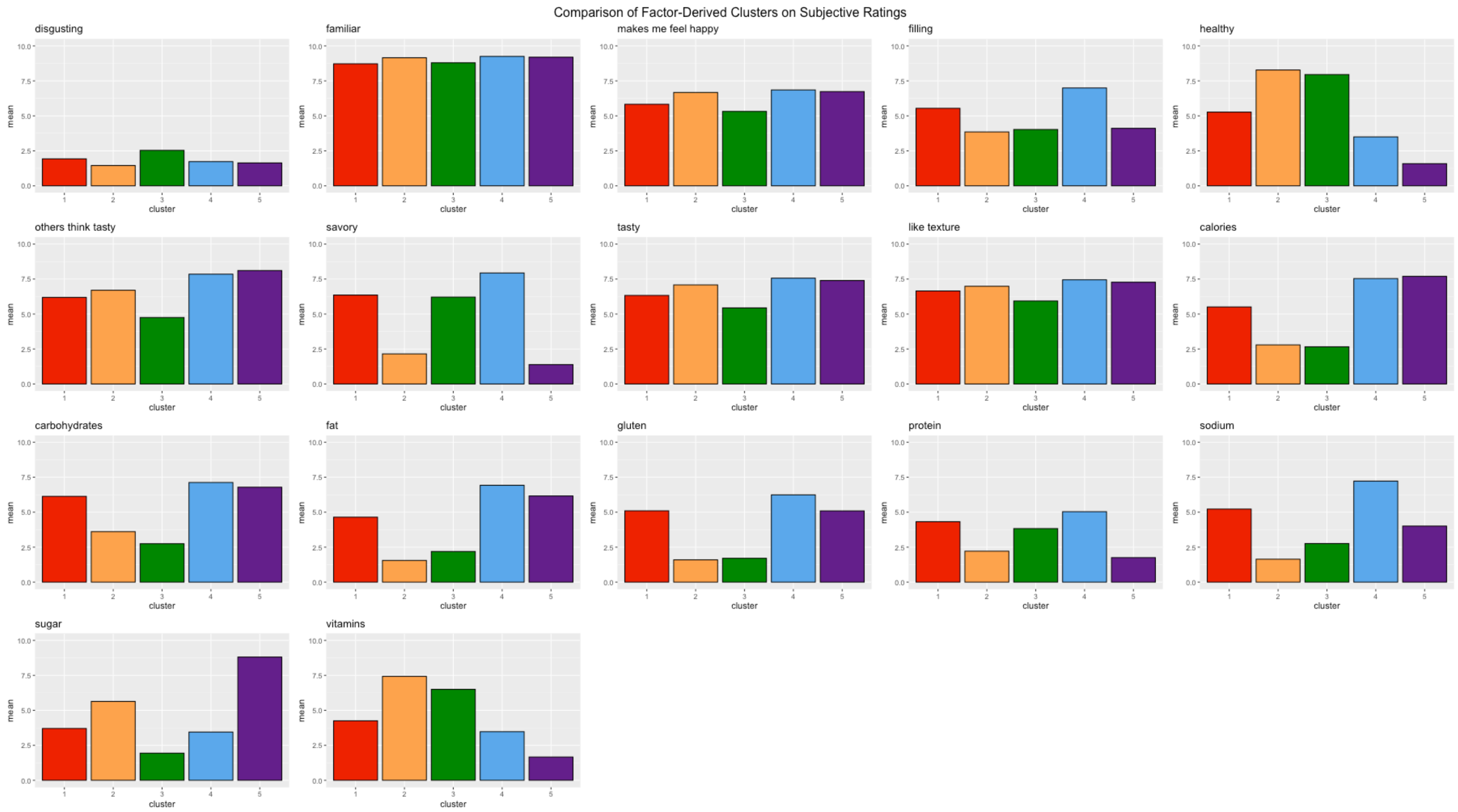

Figure 4: Mean Subjective Ratings for Foods by Cluster

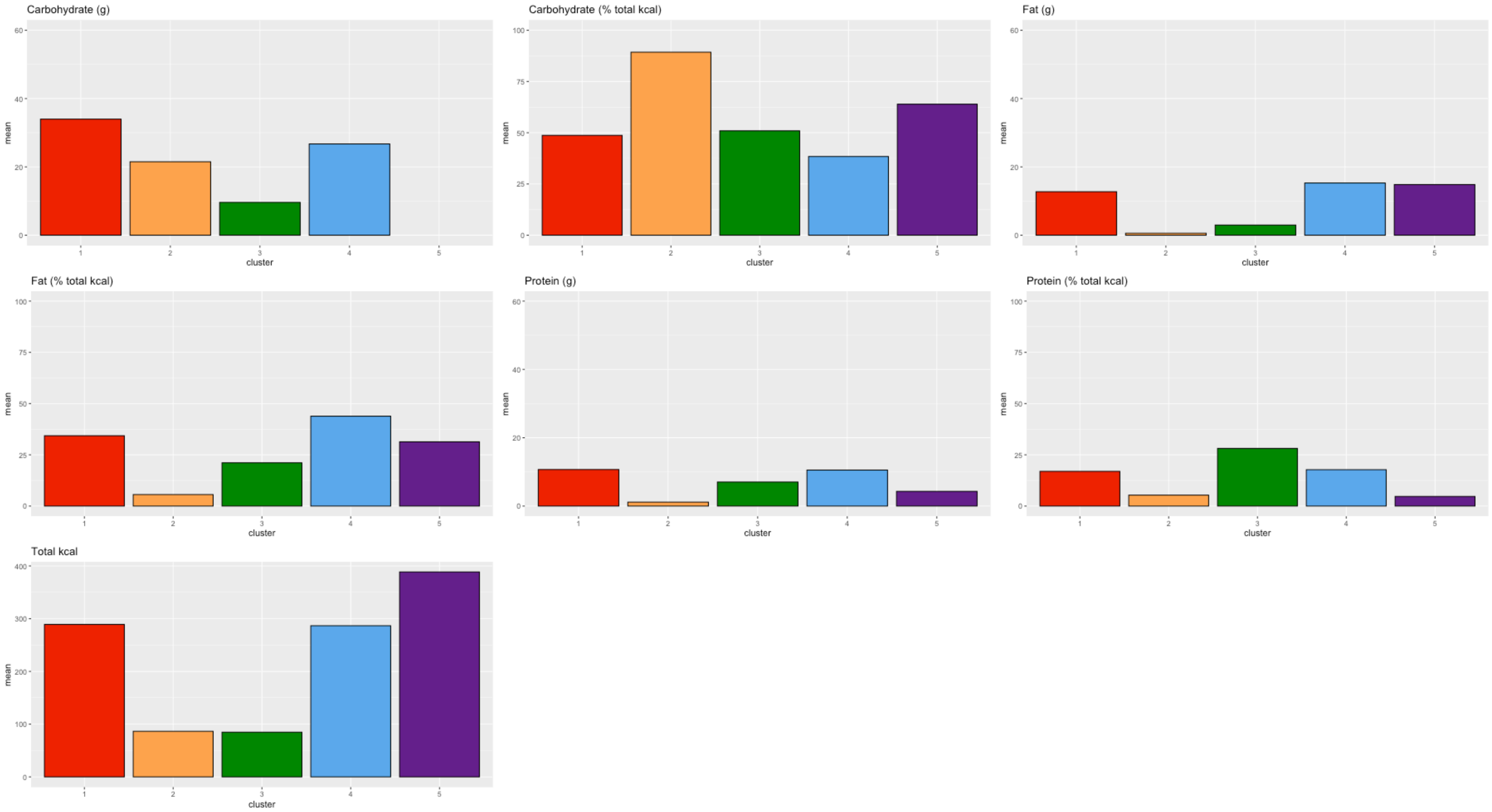

Figure 5: Mean Nutritional Characteristics for Foods by Cluster

Supplementary Information Part 2

Analyses with 76-item stimulus subset that has been used as part of the Food Choice Task (Steinglass et al., 2015)

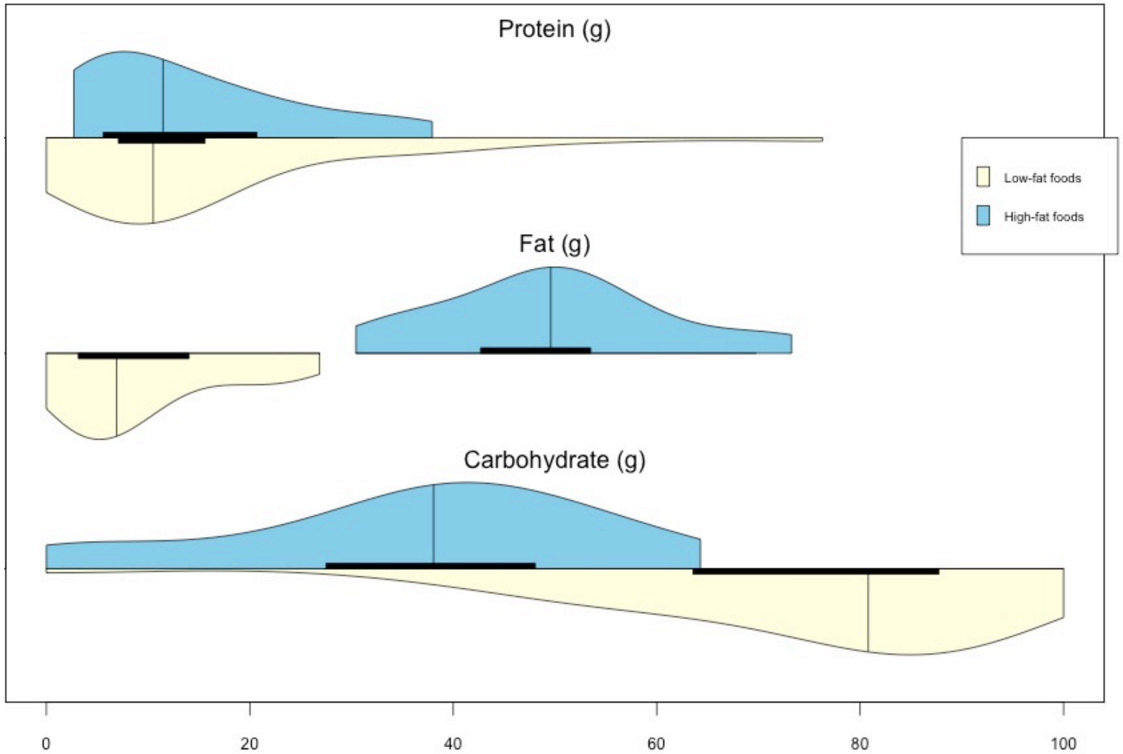

Figure 1: Probability distribution plot showing nutrient content across items of the stimulus set (grams)

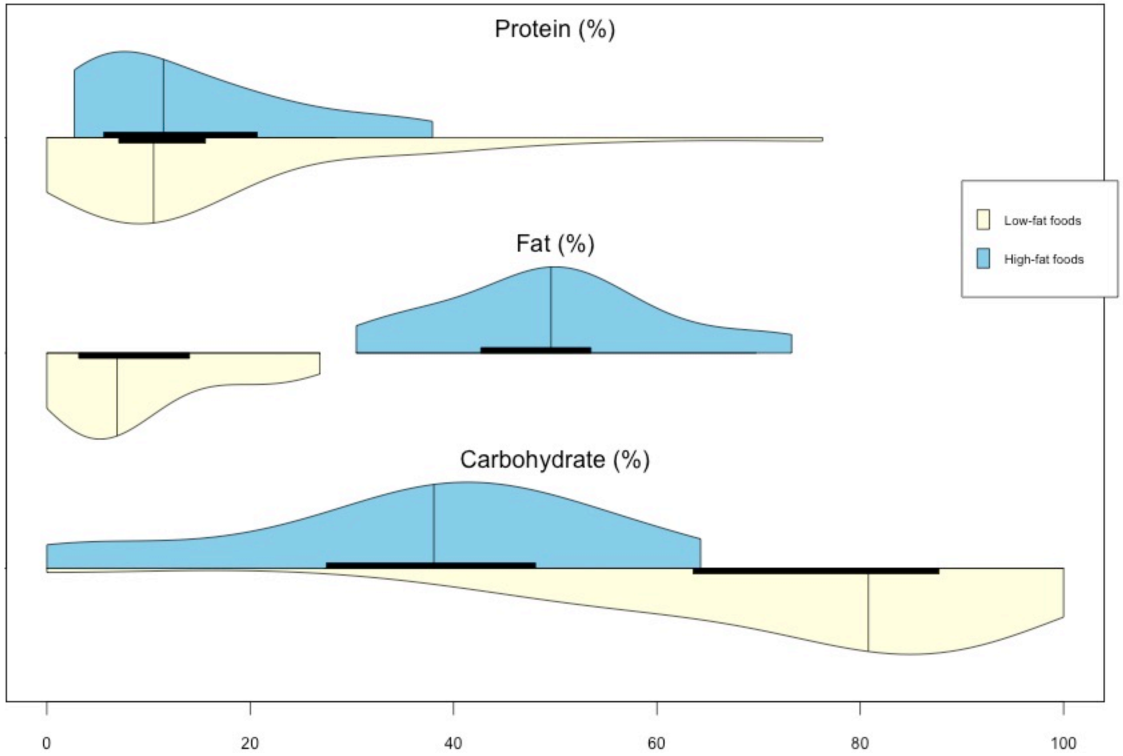

Figure 2: Probability distribution plot showing nutrient content across items of the stimulus set (as % of total calories)

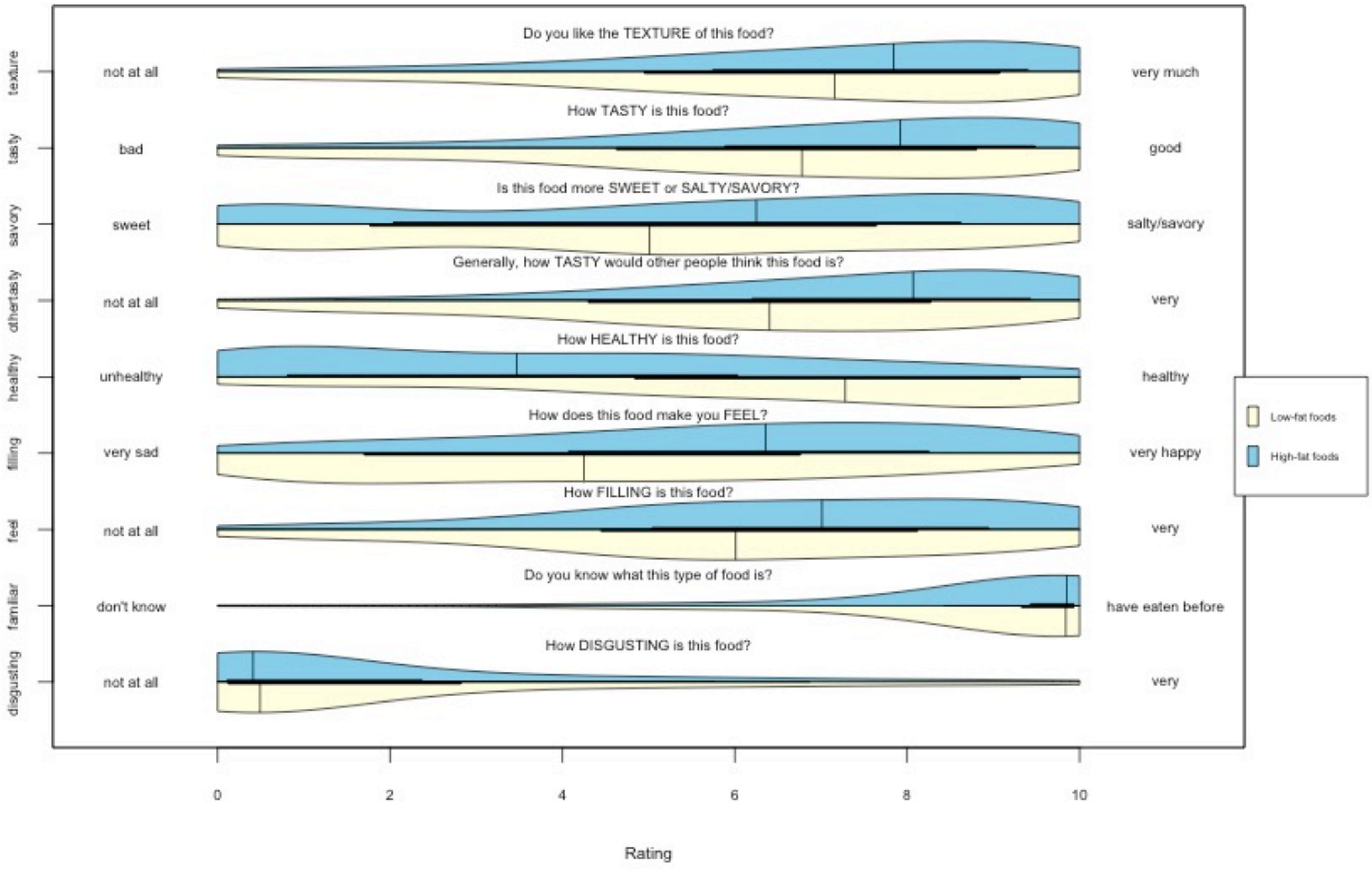

Figure 3: Probability distribution plot of food characteristic ratings

Plots show the distribution of scores for each food characteristic rating across the collection of low-fat (lower/yellow) and high-fat (upper/blue) foods of the original Food Choice Task. Every rating (for each of the Food Choice Task food-items, from all 1054 participants) is included. N ratings for low-fat foods = 13,284; n ratings for high-fat foods = 13,999.

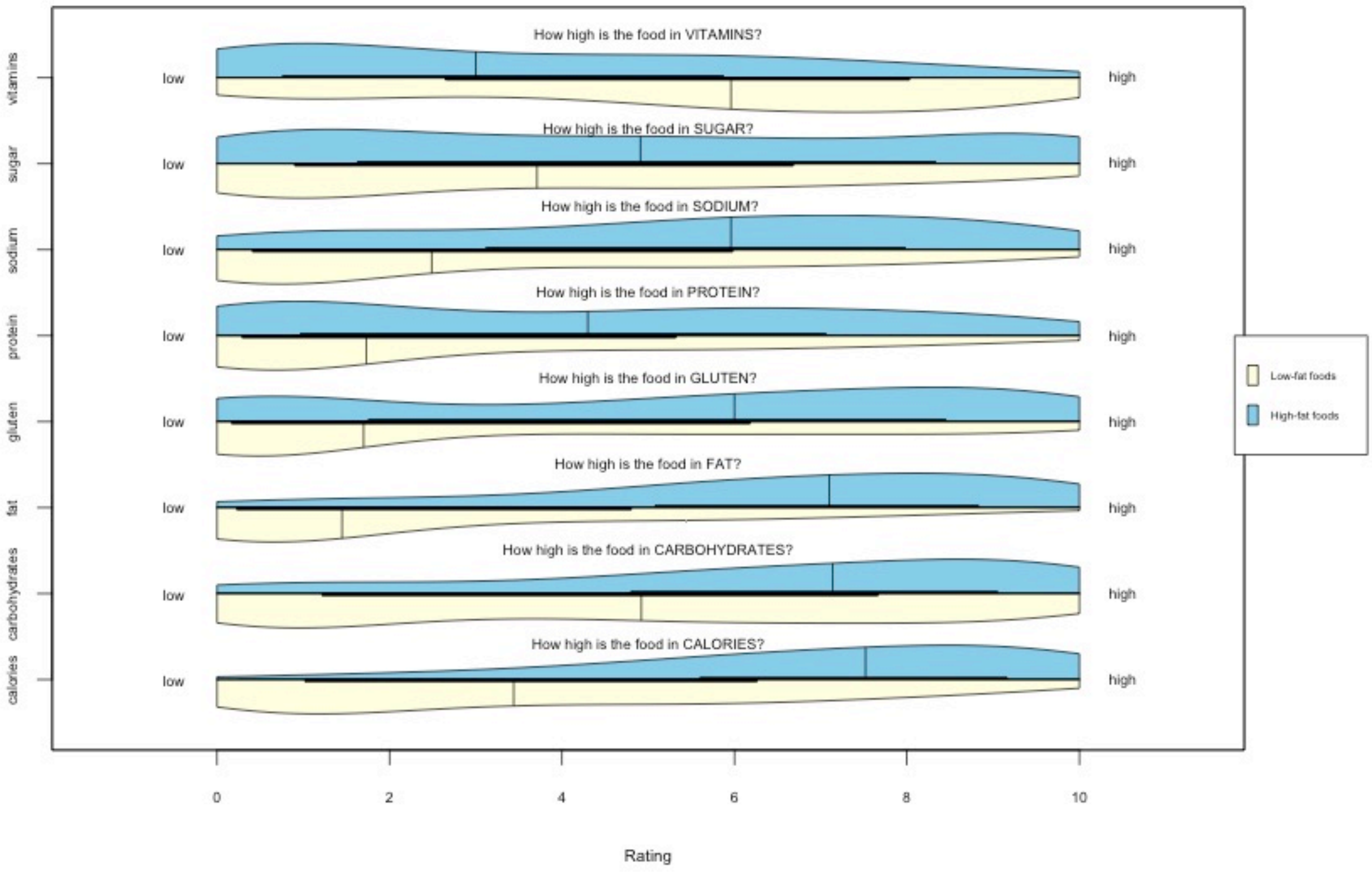

Figure 4: Probability distribution plot of estimated nutritional content ratings

Plots show the distribution of scores for each rating of nutritional content across the collection of low-fat (lower/yellow) and high-fat (upper/blue) foods of the original Food Choice Task. Every rating (for each of Food Choice Task food-items, from all 1054 participants) is included. N ratings for low-fat foods = 13,284; n ratings for high-fat foods = 13,999.

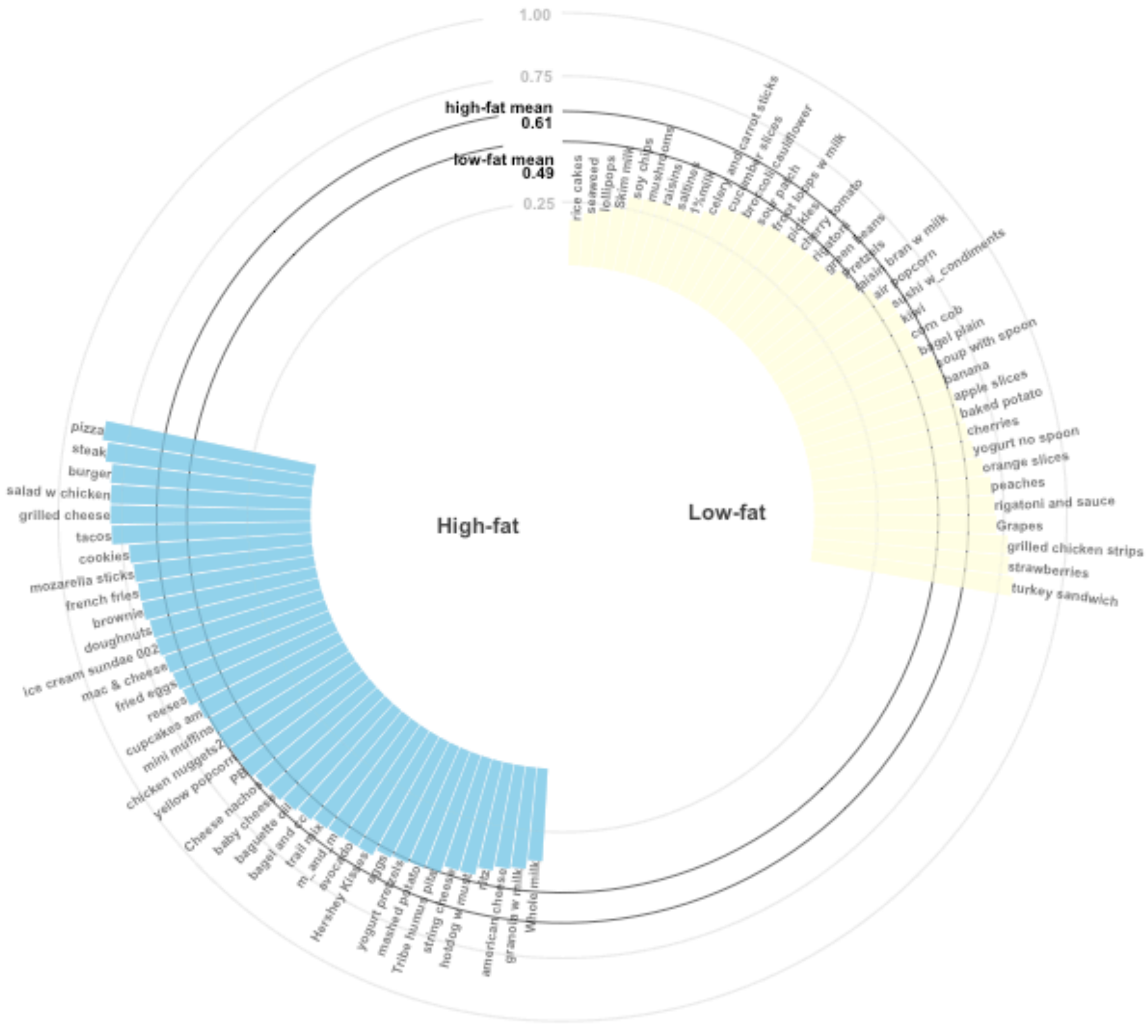

Figure 5: Selection of Each Food in Choice Phase

The proportion of times each food was selected over the reference item in the choice phase was calculated across participants.

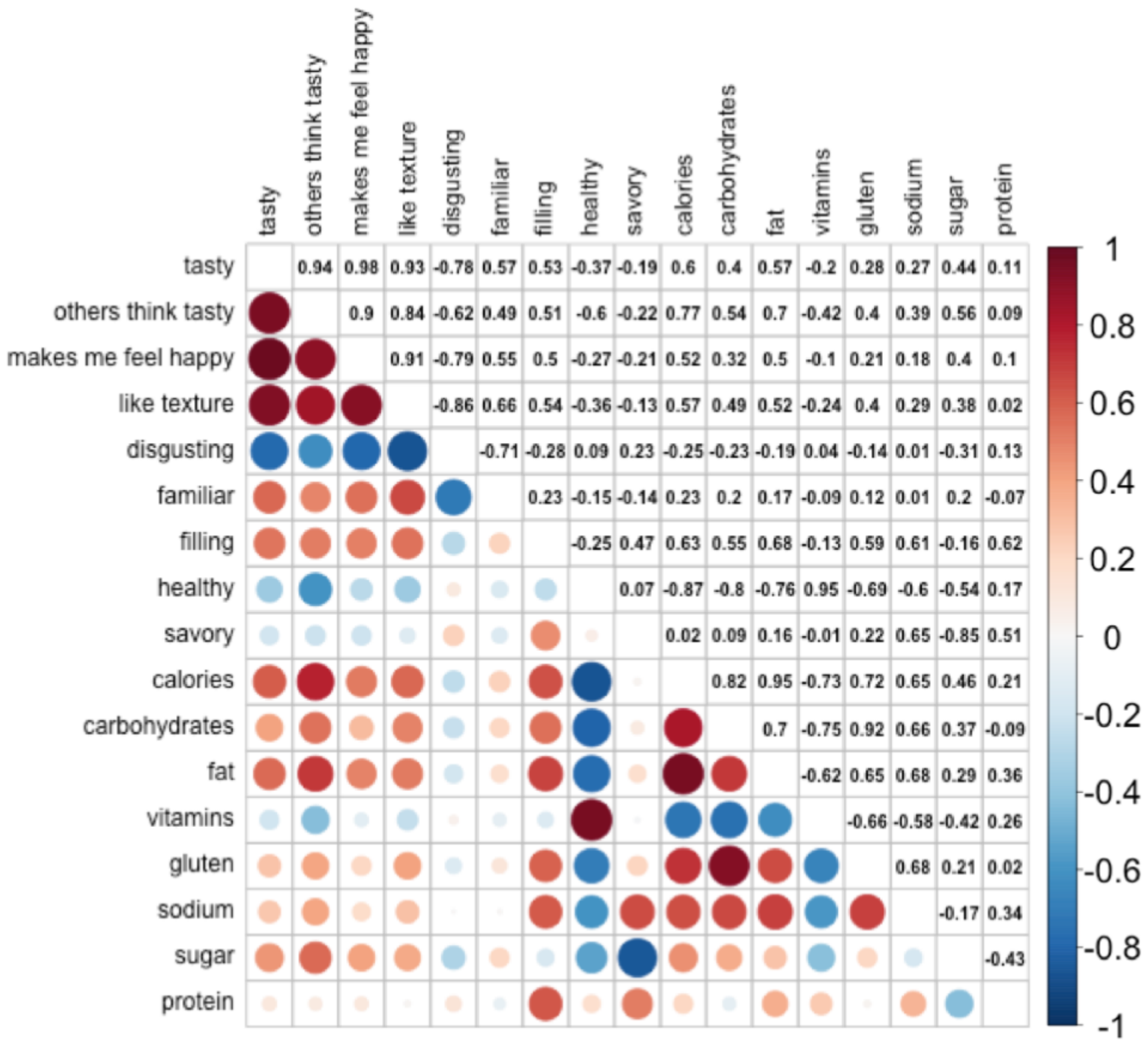

Figure 6: Correlations between subjective rating dimensions across stimulus set

The matrix shows Pearson R correlations (described using a heatmap function and numeric values), which index the strength of association, between each pair of attribute ratings. The mean for each attribute rating, for each food, was calculated, using all available participant ratings. Correlations between average ratings across all foods were then determined.

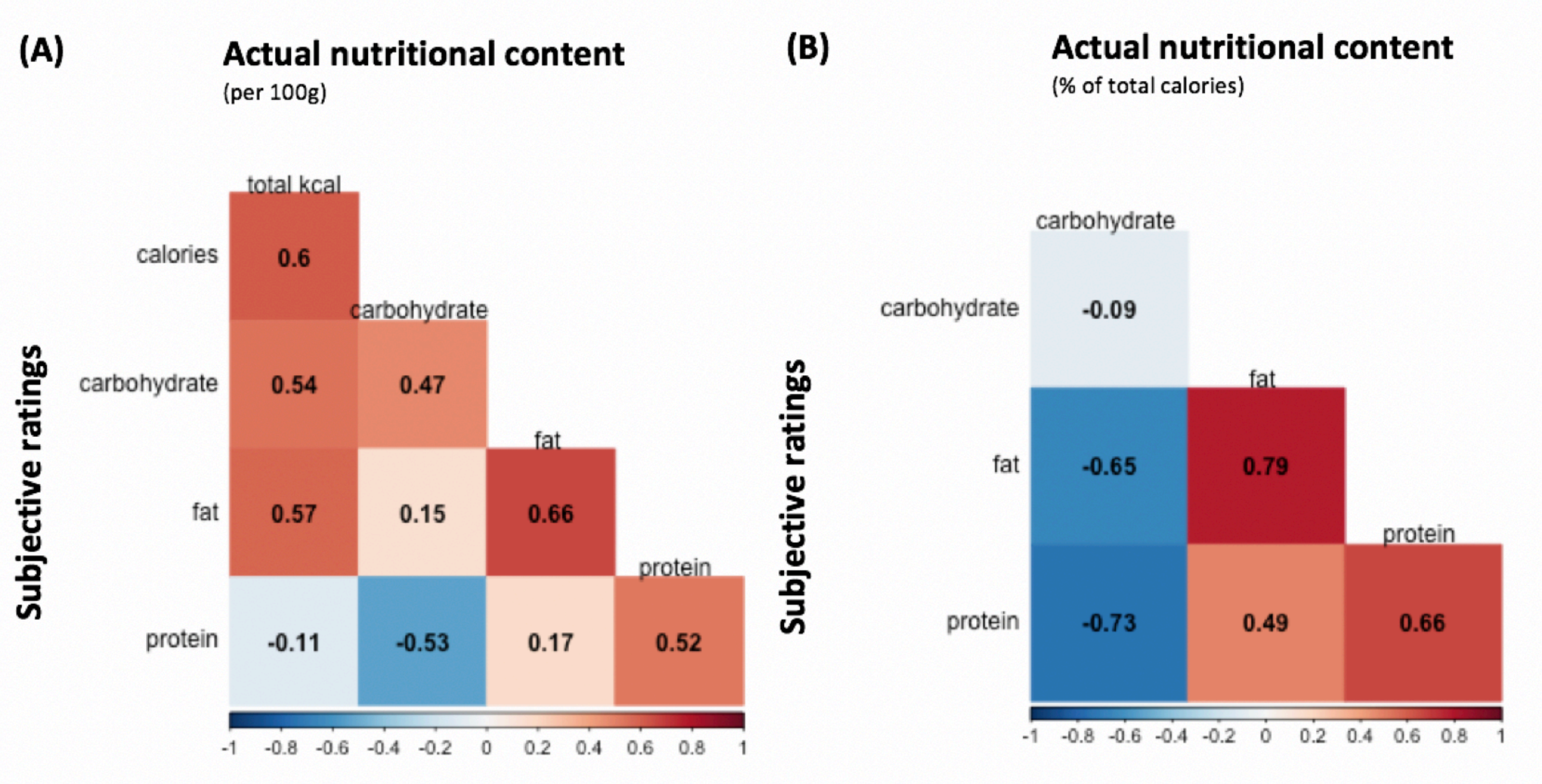

Figure 7: Correlation between subjective ratings and actual nutritional content

The correlations between average participant ratings for calorie, carbohydrate, fat and protein content, and actual nutrient content values, were estimated across the 76 food-items. (A) Pearson correlation coefficients reflecting the association between participant ratings of nutrient content and actual calorie/macronutrient content per 100g. (B) Pearson correlation coefficients reflecting the association between participant ratings of nutrient content and actual macronutrient content as a percentage of total calories.

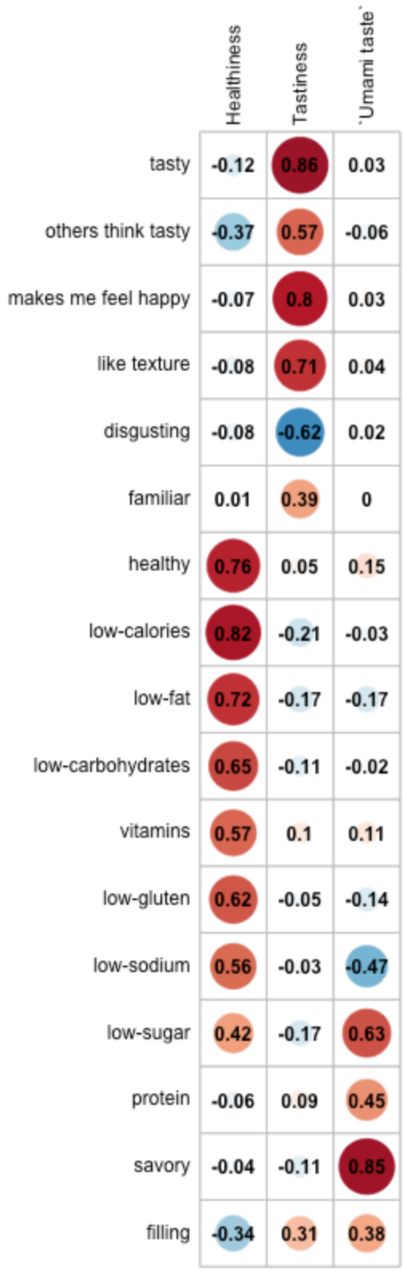

Figure 8: Loadings of the rating attributes on the three latent factors

Ratings for calories, fat, carbohydrates, gluten, sodium and sugar were reversed so that higher ratings indicated lower content of these nutrients. The corresponding attributes were relabeled accordingly (e.g. calories became low calories).

Factor 1 was labelled as Healthiness given high positive loadings from low-calories, low-carbohydrates, low-fat, low-gluten, and low-sodium, content attributes, and positive loadings from healthiness and vitamin content ratings. Factor 2 was labelled Tastiness based on positive loadings from ratings of tastiness, estimates of others' tastiness evaluations, favourable texture, feelings of happiness evoked from the food, and negative loadings from disgust ratings. Factor 3 was labelled as an umami taste factor, given the high positive loading of savoriness ratings, and (to a lesser extent) ratings of protein and low-sugar content.

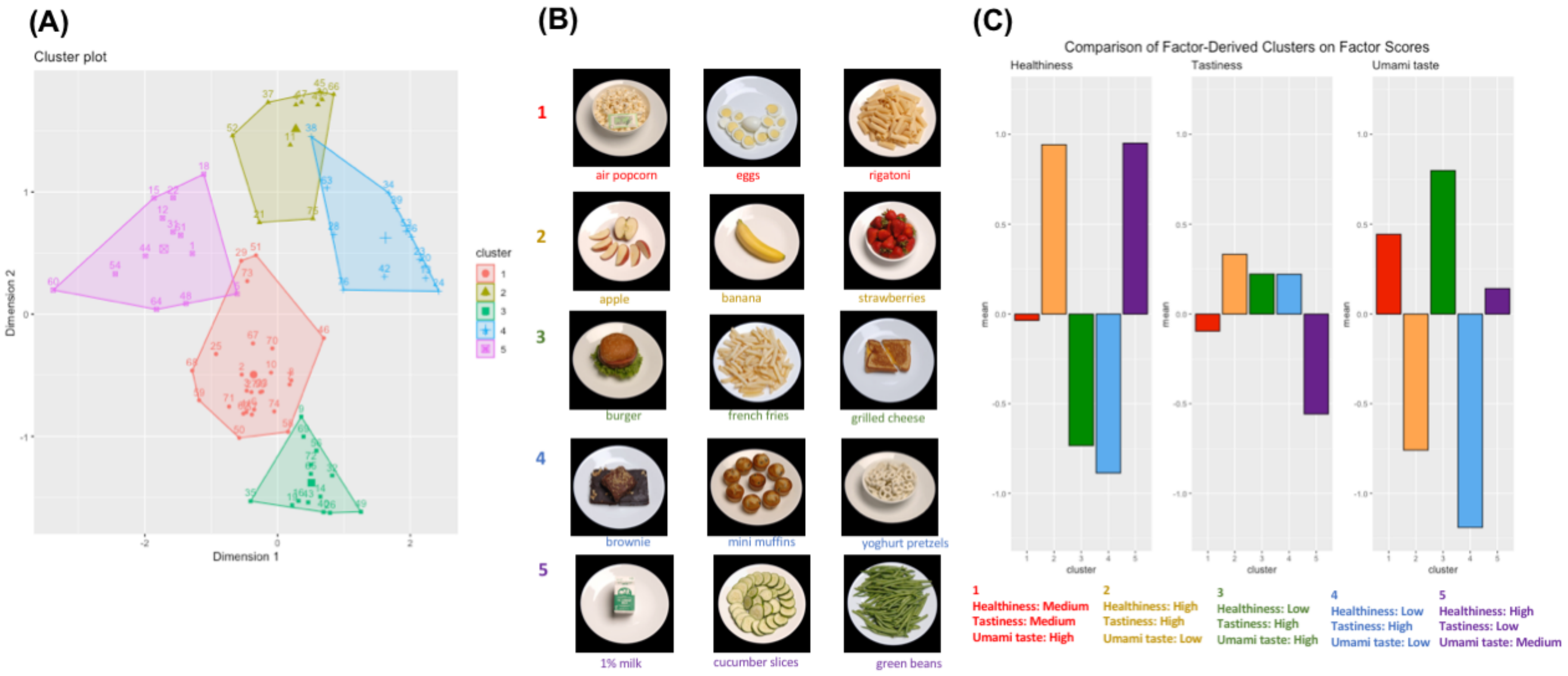

Figure 9: Results of Cluster Analysis of Food Choice Task Foods based on Latent Factor Scores

Three latent factor scores (reflecting healthiness, tastiness and umami taste) were calculated for each food-item, using Thompson's regression method and ratings for each of the 17 rated attributes. (A) Foods were clustered into five categories based on their average factor scores. (B) Examples of food-items within each cluster. (C) Mean factor scores for each cluster of foods were calculated, using all participant data (i.e. each participant provided three factor scores for each food-item in their set that was included in the original Food Choice Task), demonstrating the five clusters varied on the latent dimensions of healthiness, tastiness and umami taste (C).

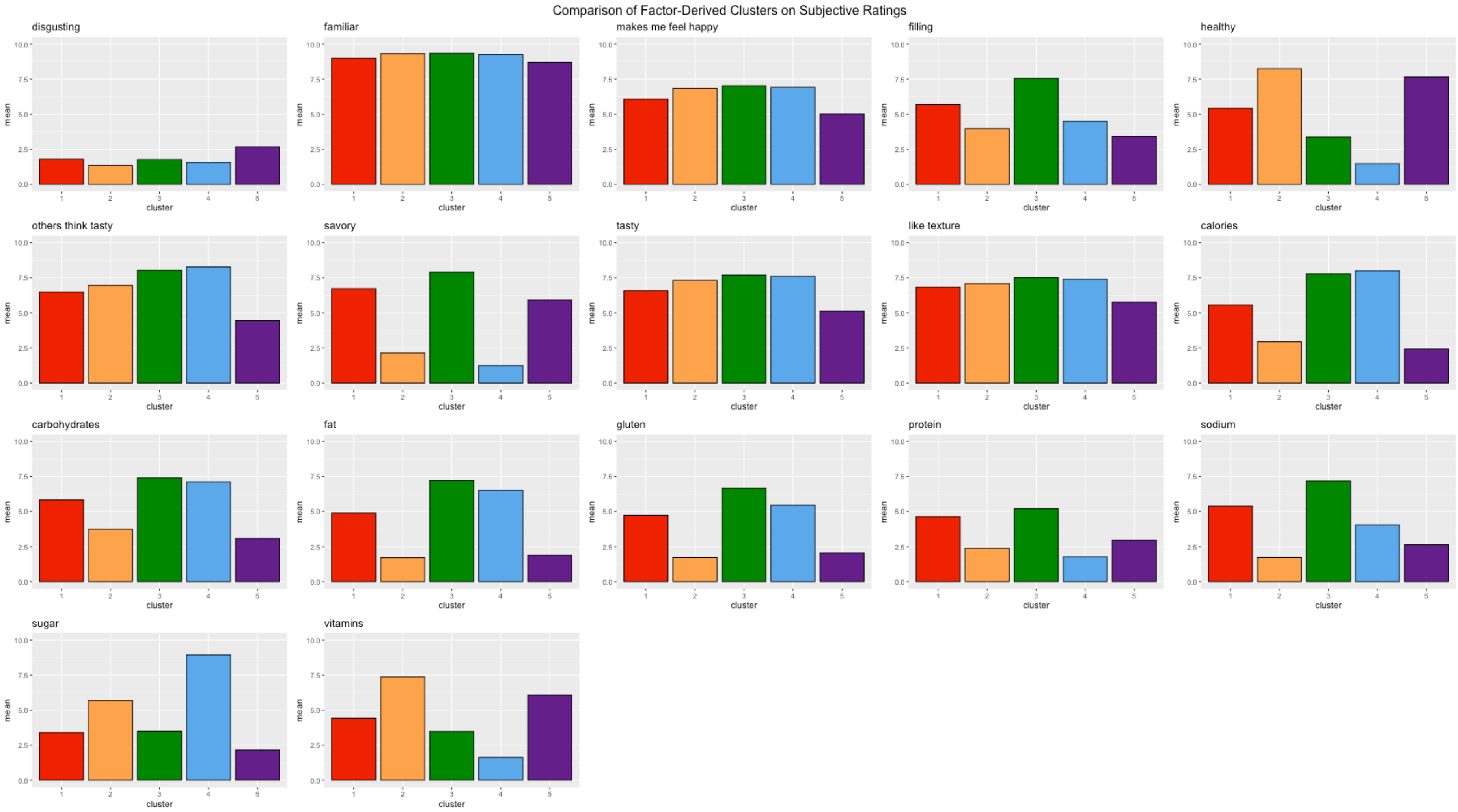

Figure 10: Mean Subjective Ratings for Foods in each Cluster

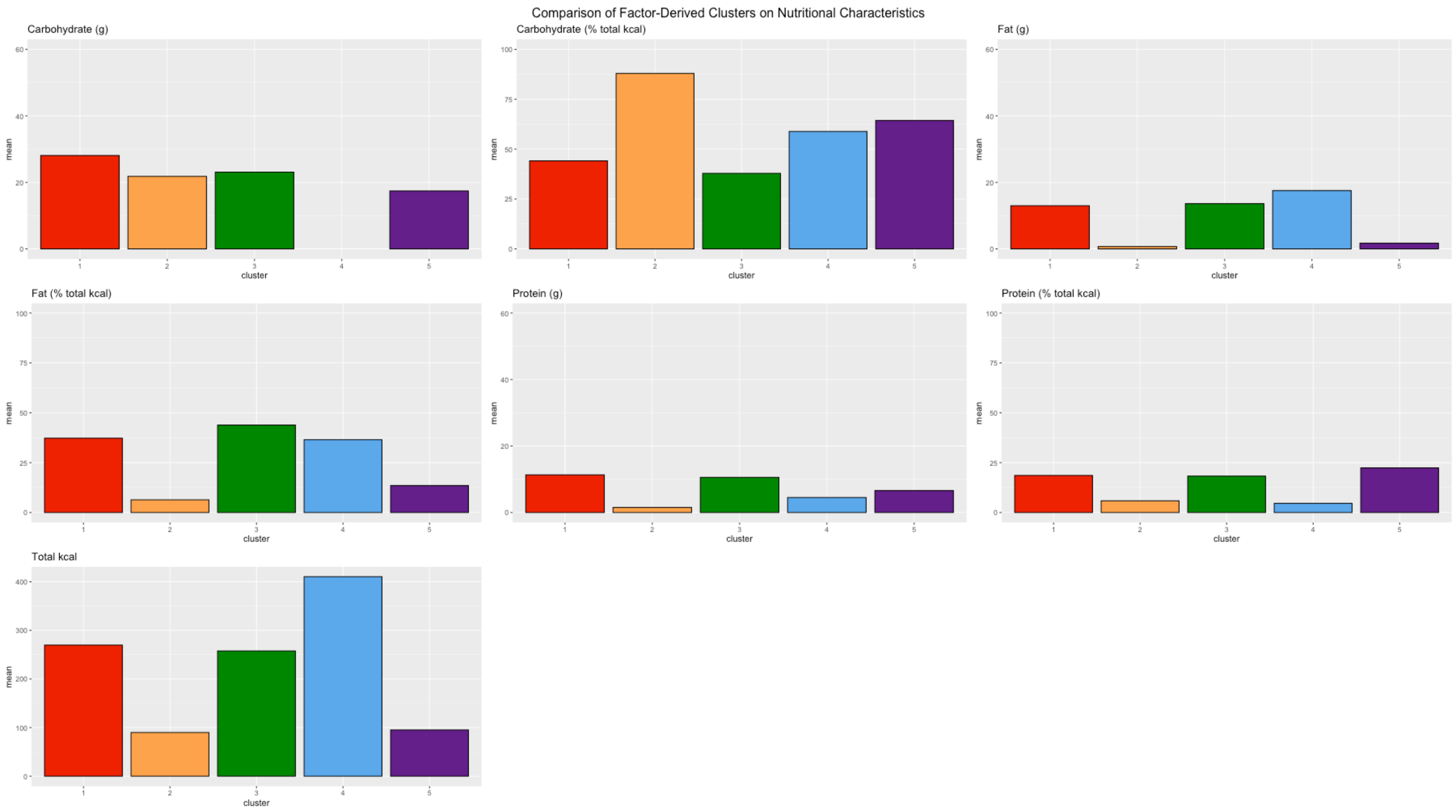

Figure 11: Mean Nutritional Information for Foods in each Cluster

References

Steinglass, J., Foerde, K., Kostro, K., Shohamy, D., & Timothy Walsh, B. (2015). Restrictive Food Intake As A Choice – A Paradigm for Study. *Int J Eat Disord, 48*(1), 59-66. doi:10.1002/eat.22345
