# Supplementary figures and images for: Food Folio by Columbia Center for Eating Disorders: A Freely Available Food Image Database (part 2 of 2)
Source: Front Psychol. 2020 Dec 23;11:585044. doi: 10.3389/fpsyg.2020.585044 (PMC7785939; doi:10.3389/fpsyg.2020.585044)

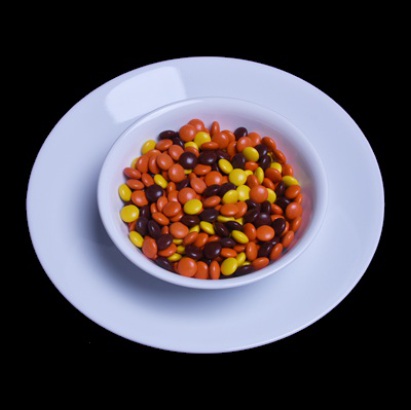

Supplement: Supplementary file 4 [file Data_Sheet_2.ZIP › reesespieces.jpg]

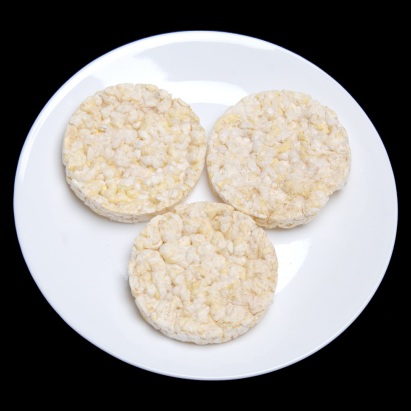

Supplement: Supplementary file 4 [file Data_Sheet_2.ZIP › rice cakes.jpg]

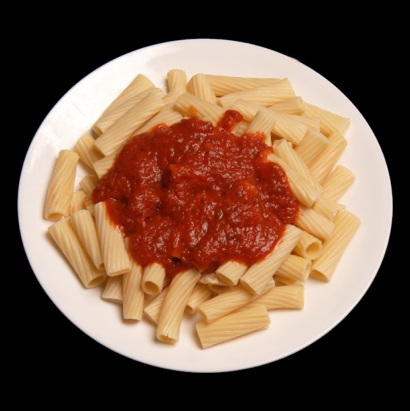

Supplement: Supplementary file 4 [file Data_Sheet_2.ZIP › rigatoni and sauce.jpg]

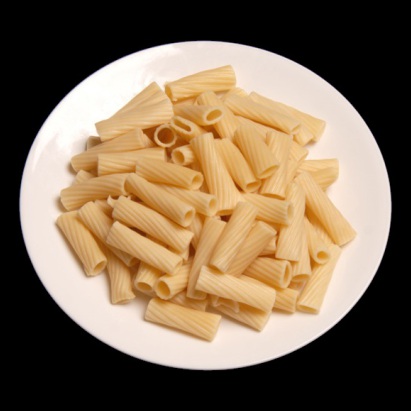

Supplement: Supplementary file 4 [file Data_Sheet_2.ZIP › rigatoni.jpg]

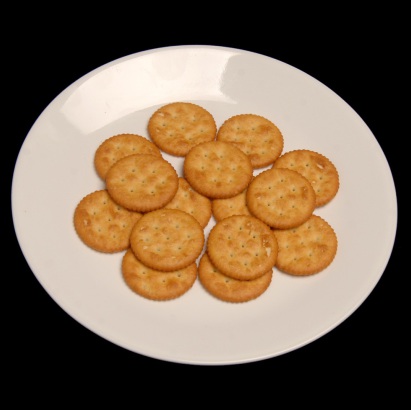

Supplement: Supplementary file 4 [file Data_Sheet_2.ZIP › ritz.jpg]

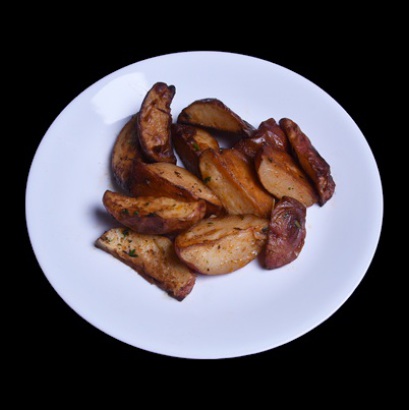

Supplement: Supplementary file 4 [file Data_Sheet_2.ZIP › roastedpotatoes.jpg]

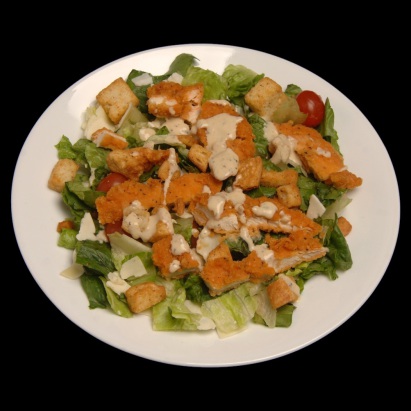

Supplement: Supplementary file 4 [file Data_Sheet_2.ZIP › salad w chicken.jpg]

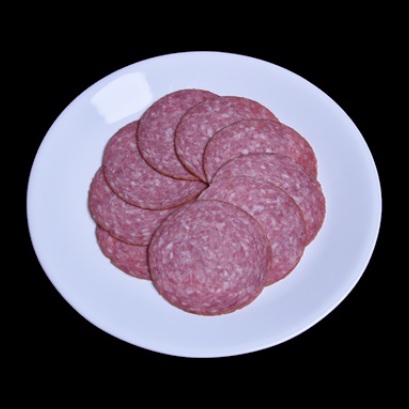

Supplement: Supplementary file 4 [file Data_Sheet_2.ZIP › salami.jpg]

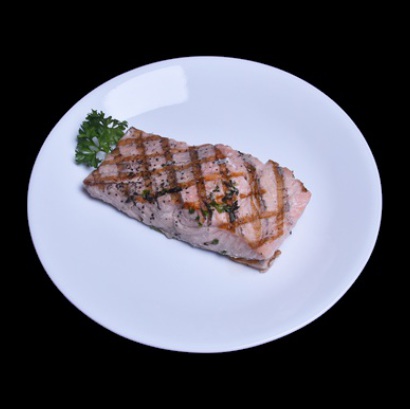

Supplement: Supplementary file 4 [file Data_Sheet_2.ZIP › salmon.jpg]

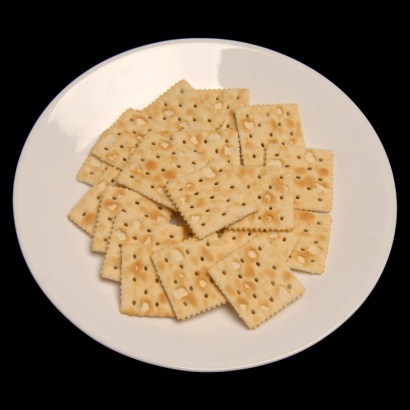

Supplement: Supplementary file 4 [file Data_Sheet_2.ZIP › saltines.jpg]

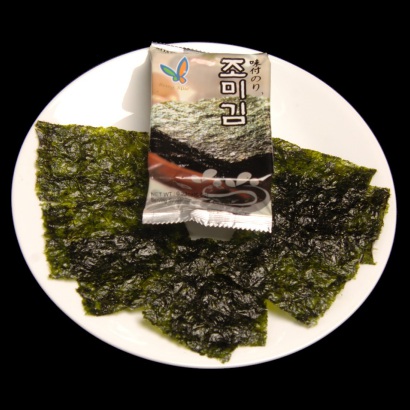

Supplement: Supplementary file 4 [file Data_Sheet_2.ZIP › seaweed.jpg]

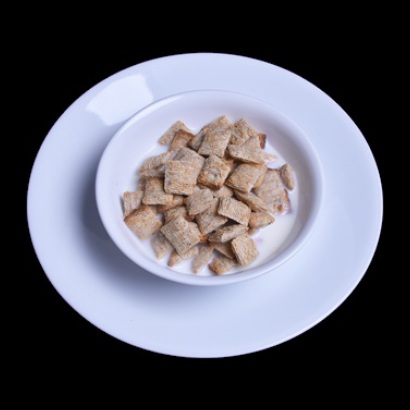

Supplement: Supplementary file 4 [file Data_Sheet_2.ZIP › shreddedwheatwithmilk.jpg]

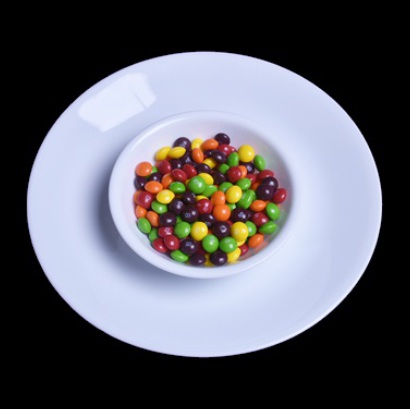

Supplement: Supplementary file 4 [file Data_Sheet_2.ZIP › skittles.jpg]

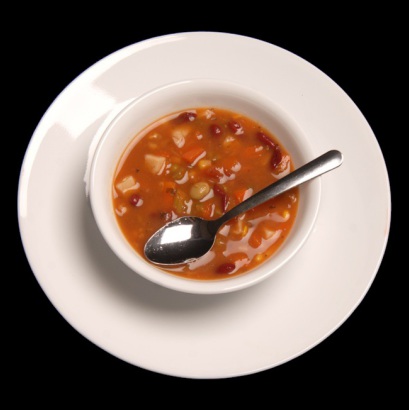

Supplement: Supplementary file 4 [file Data_Sheet_2.ZIP › soup with spoon.jpg]

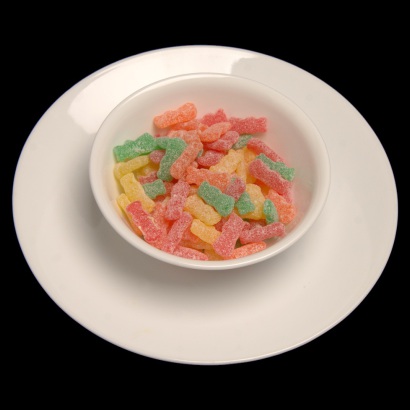

Supplement: Supplementary file 4 [file Data_Sheet_2.ZIP › sour patch.jpg]

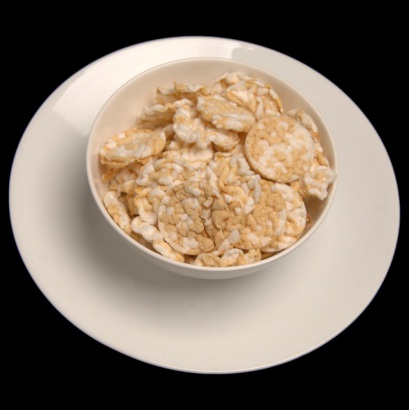

Supplement: Supplementary file 4 [file Data_Sheet_2.ZIP › soy chips.jpg]

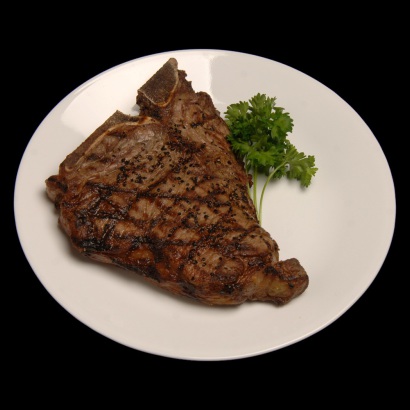

Supplement: Supplementary file 4 [file Data_Sheet_2.ZIP › steak.jpg]

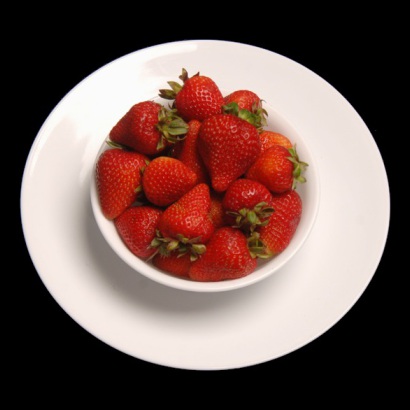

Supplement: Supplementary file 4 [file Data_Sheet_2.ZIP › strawberries.jpg]

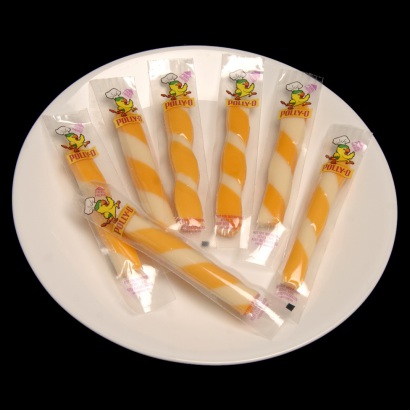

Supplement: Supplementary file 4 [file Data_Sheet_2.ZIP › string cheese.jpg]

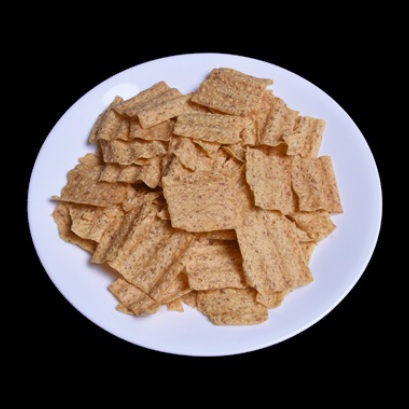

Supplement: Supplementary file 4 [file Data_Sheet_2.ZIP › sunchips.jpg]

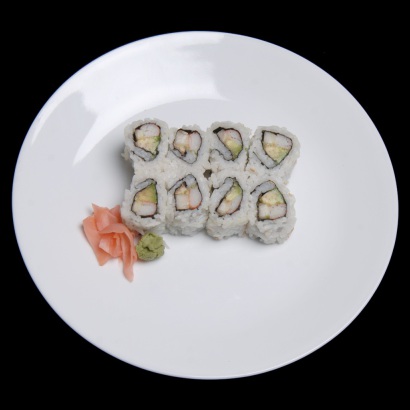

Supplement: Supplementary file 4 [file Data_Sheet_2.ZIP › sushi w_condiments.jpg]

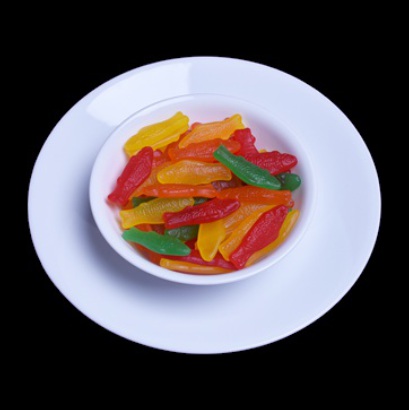

Supplement: Supplementary file 4 [file Data_Sheet_2.ZIP › swedishfish.jpg]

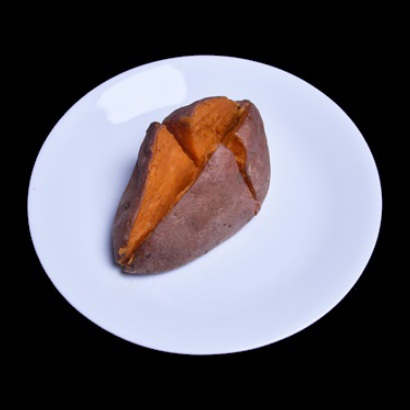

Supplement: Supplementary file 4 [file Data_Sheet_2.ZIP › sweetpotato.jpg]

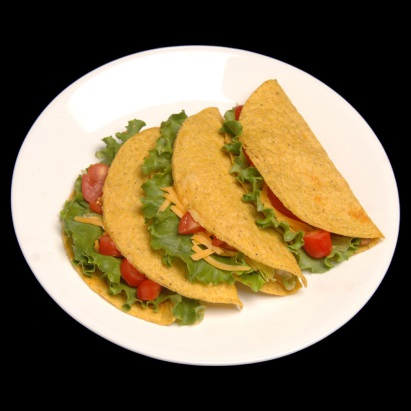

Supplement: Supplementary file 4 [file Data_Sheet_2.ZIP › tacos.jpg]

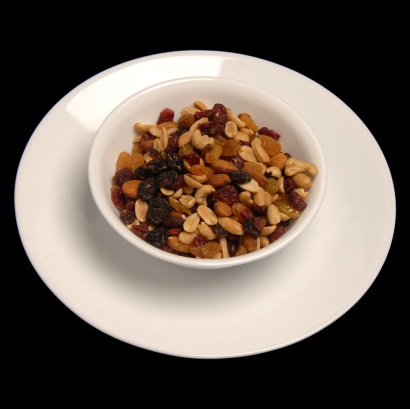

Supplement: Supplementary file 4 [file Data_Sheet_2.ZIP › trail mix.jpg]

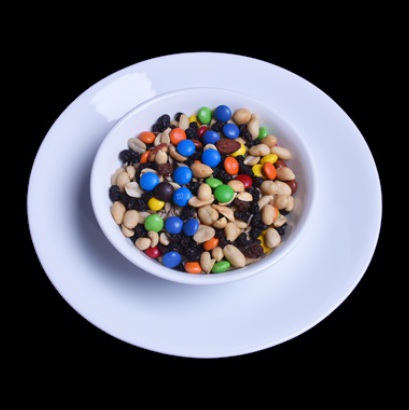

Supplement: Supplementary file 4 [file Data_Sheet_2.ZIP › trailmix.jpg]

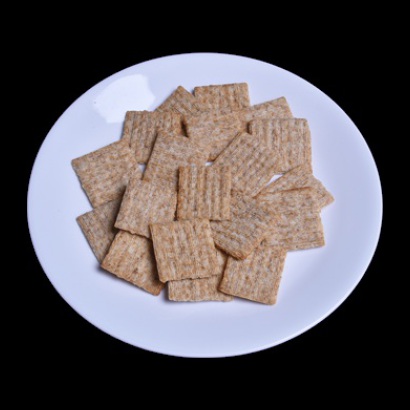

Supplement: Supplementary file 4 [file Data_Sheet_2.ZIP › triscuits.jpg]

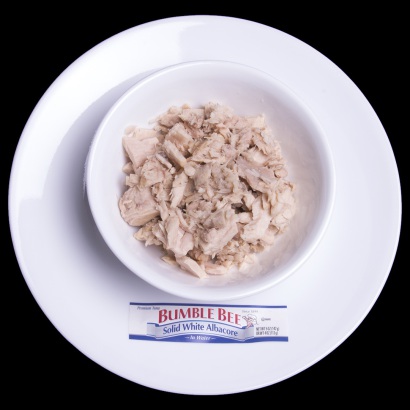

Supplement: Supplementary file 4 [file Data_Sheet_2.ZIP › tuna.jpg]

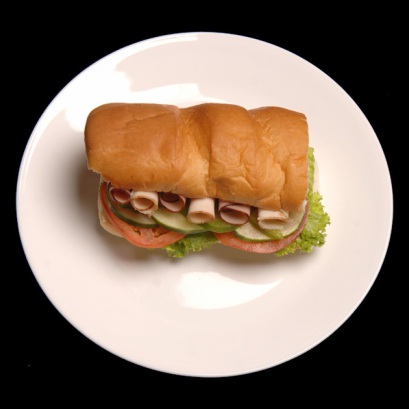

Supplement: Supplementary file 4 [file Data_Sheet_2.ZIP › turkey sandwich.jpg]

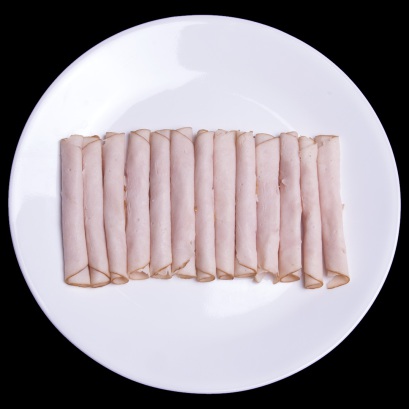

Supplement: Supplementary file 4 [file Data_Sheet_2.ZIP › turkeydelimeat.jpg]

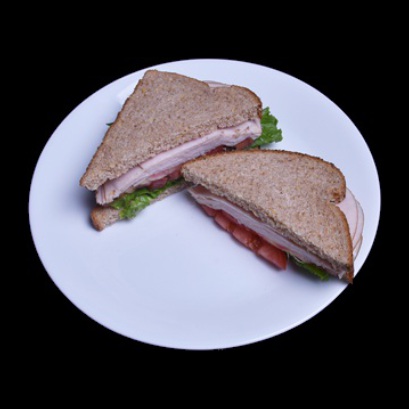

Supplement: Supplementary file 4 [file Data_Sheet_2.ZIP › turkeysandwich.jpg]

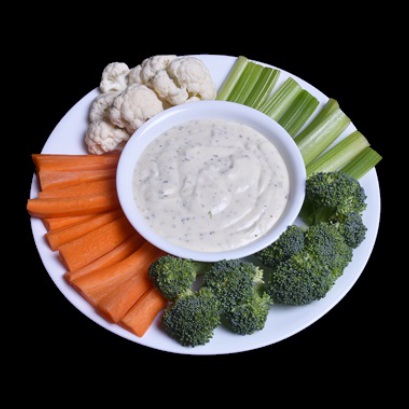

Supplement: Supplementary file 4 [file Data_Sheet_2.ZIP › veggieswithdip.jpg]

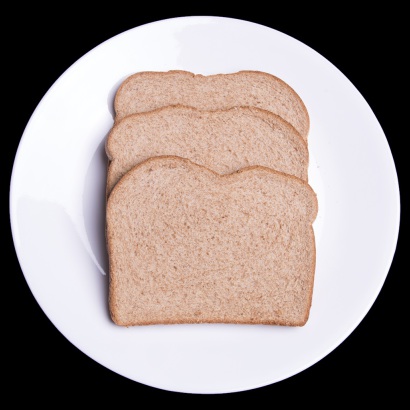

Supplement: Supplementary file 4 [file Data_Sheet_2.ZIP › wheatbread.jpg]

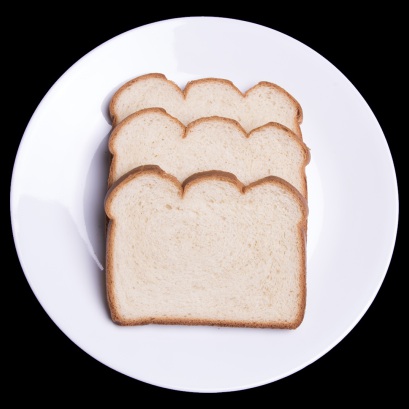

Supplement: Supplementary file 4 [file Data_Sheet_2.ZIP › whitebread.jpg]

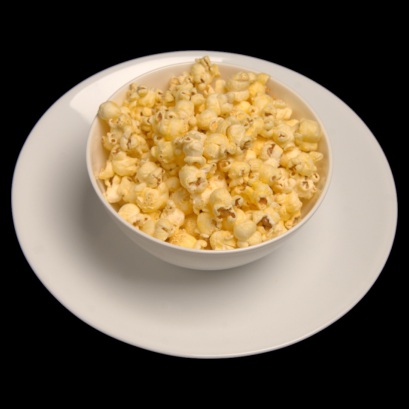

Supplement: Supplementary file 4 [file Data_Sheet_2.ZIP › yellow popcorn.jpg]

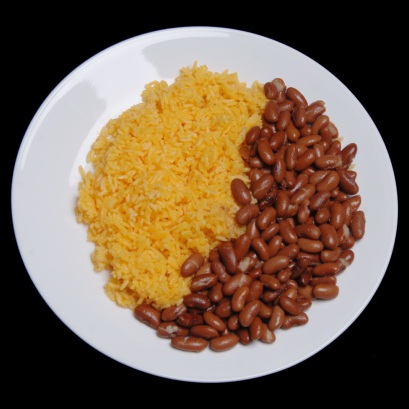

Supplement: Supplementary file 4 [file Data_Sheet_2.ZIP › yellow rice_beans.jpg]

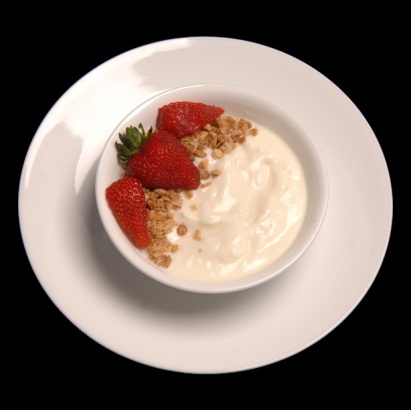

Supplement: Supplementary file 4 [file Data_Sheet_2.ZIP › yogurt no spoon.jpg]

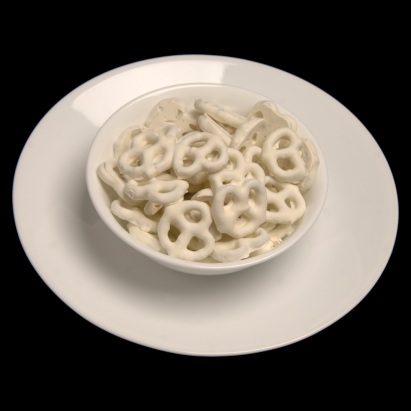

Supplement: Supplementary file 4 [file Data_Sheet_2.ZIP › yogurt pretzels.jpg]
