# Supplementary figures and images for: Food Folio by Columbia Center for Eating Disorders: A Freely Available Food Image Database (part 1 of 2)
Source: Front Psychol. 2020 Dec 23;11:585044. doi: 10.3389/fpsyg.2020.585044 (PMC7785939; doi:10.3389/fpsyg.2020.585044)

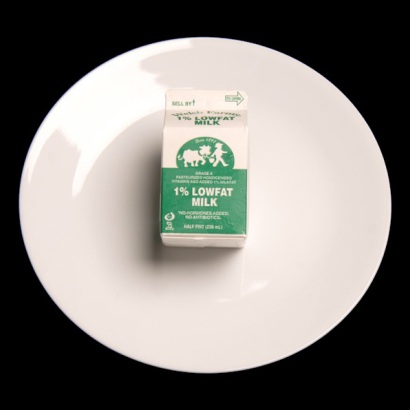

Supplement: Supplementary file 4 [file Data_Sheet_2.ZIP › 1%milk.jpg]

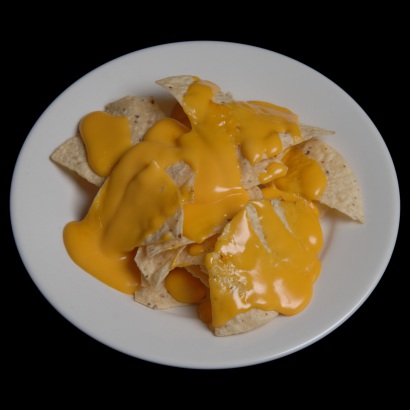

Supplement: Supplementary file 4 [file Data_Sheet_2.ZIP › Cheese nachos.jpg]

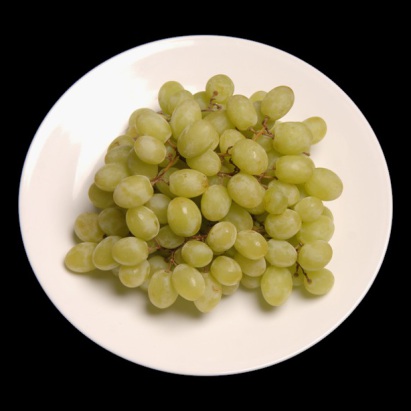

Supplement: Supplementary file 4 [file Data_Sheet_2.ZIP › Grapes.jpg]

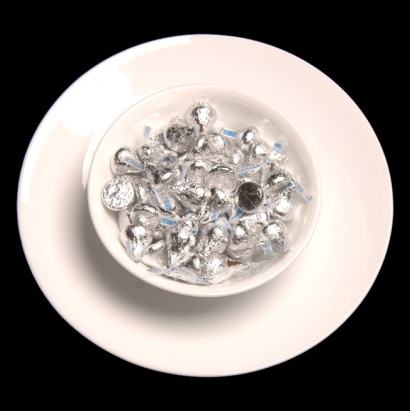

Supplement: Supplementary file 4 [file Data_Sheet_2.ZIP › Hershey Kisses.jpg]

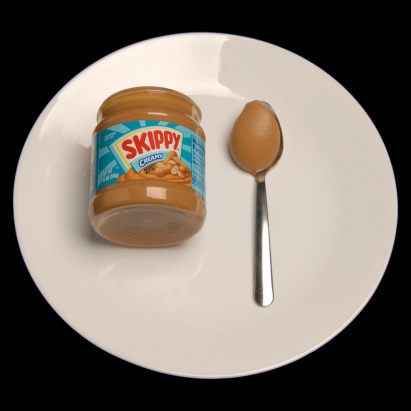

Supplement: Supplementary file 4 [file Data_Sheet_2.ZIP › PB.jpg]

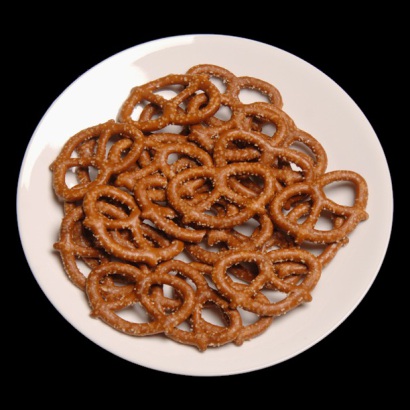

Supplement: Supplementary file 4 [file Data_Sheet_2.ZIP › Pretzels.jpg]

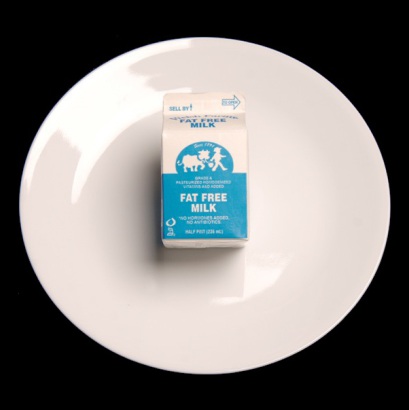

Supplement: Supplementary file 4 [file Data_Sheet_2.ZIP › Skim milk.jpg]

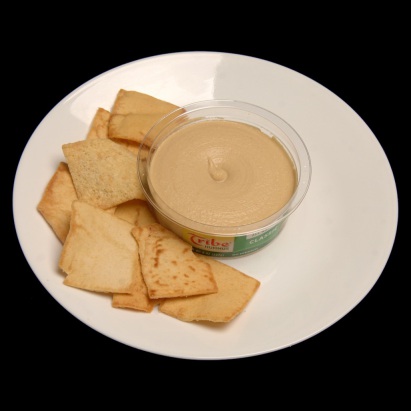

Supplement: Supplementary file 4 [file Data_Sheet_2.ZIP › Tribe humus pita.jpg]

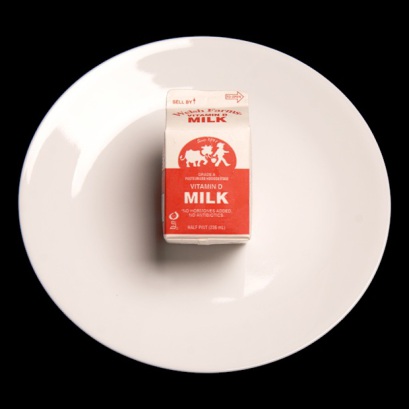

Supplement: Supplementary file 4 [file Data_Sheet_2.ZIP › Whole milk.jpg]

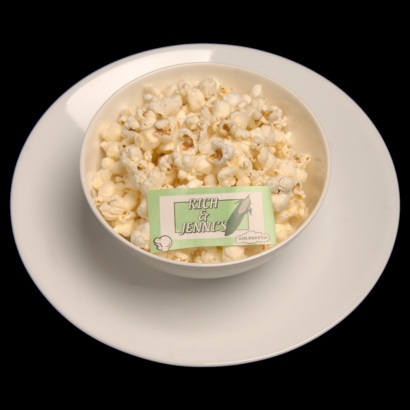

Supplement: Supplementary file 4 [file Data_Sheet_2.ZIP › air popcorn.jpg]

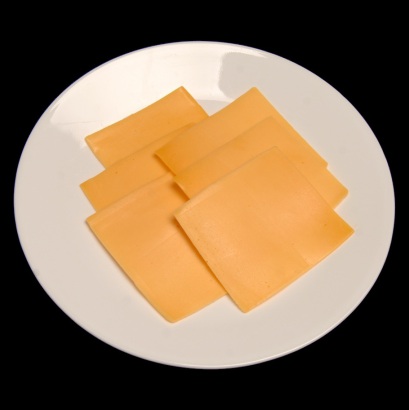

Supplement: Supplementary file 4 [file Data_Sheet_2.ZIP › american cheese.jpg]

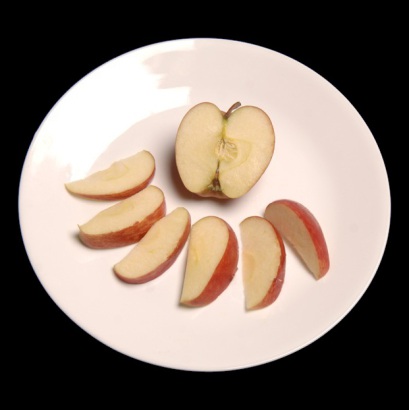

Supplement: Supplementary file 4 [file Data_Sheet_2.ZIP › apple slices.jpg]

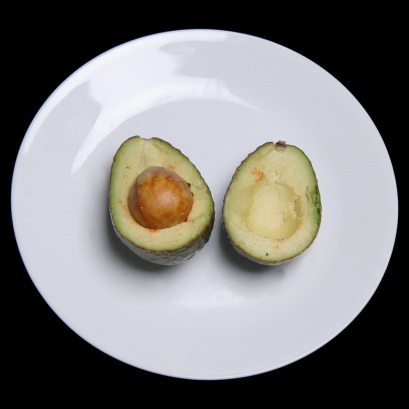

Supplement: Supplementary file 4 [file Data_Sheet_2.ZIP › avocado.jpg]

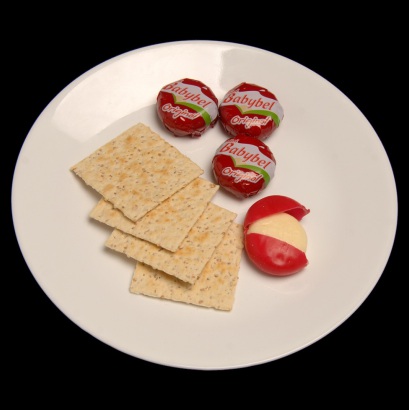

Supplement: Supplementary file 4 [file Data_Sheet_2.ZIP › baby cheese.jpg]

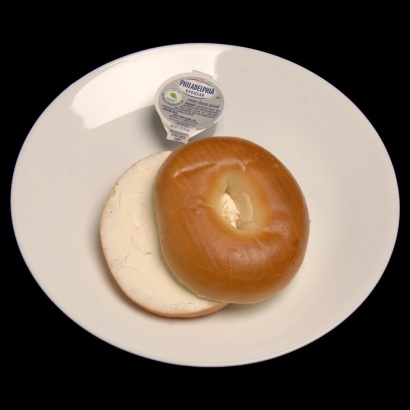

Supplement: Supplementary file 4 [file Data_Sheet_2.ZIP › bagel and cc.jpg]

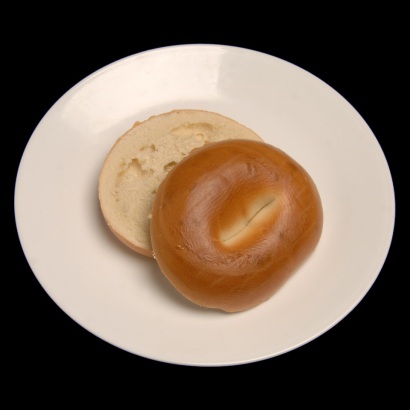

Supplement: Supplementary file 4 [file Data_Sheet_2.ZIP › bagel plain.jpg]

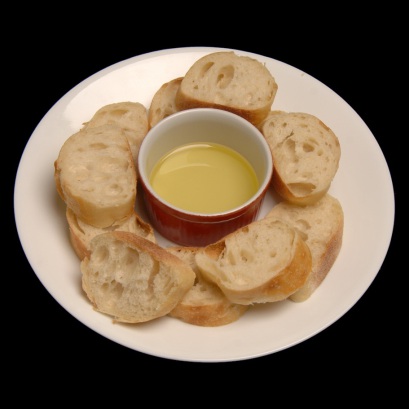

Supplement: Supplementary file 4 [file Data_Sheet_2.ZIP › baguette oil.jpg]

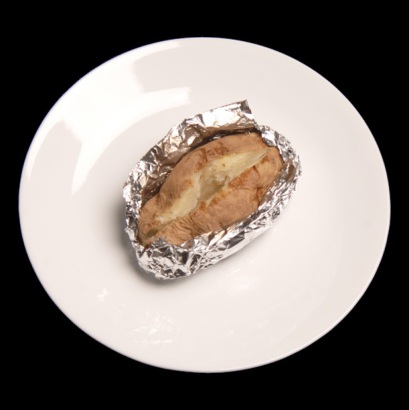

Supplement: Supplementary file 4 [file Data_Sheet_2.ZIP › baked potato.jpg]

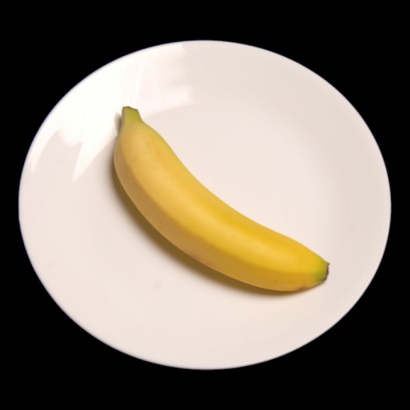

Supplement: Supplementary file 4 [file Data_Sheet_2.ZIP › banana.jpg]

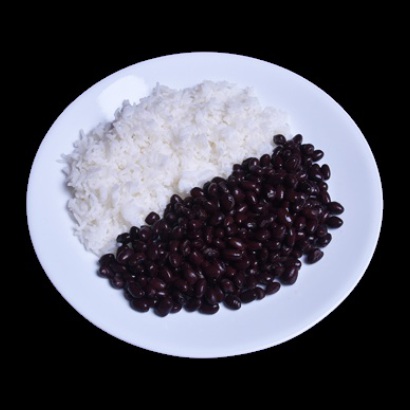

Supplement: Supplementary file 4 [file Data_Sheet_2.ZIP › blackbeanswithrice.jpg]

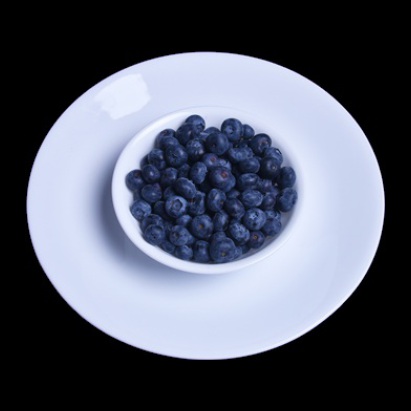

Supplement: Supplementary file 4 [file Data_Sheet_2.ZIP › blueberries.jpg]

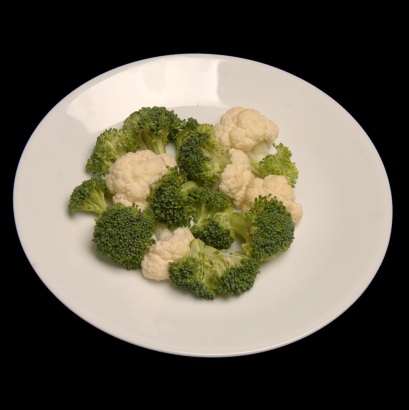

Supplement: Supplementary file 4 [file Data_Sheet_2.ZIP › broccoli cauliflower.jpg]

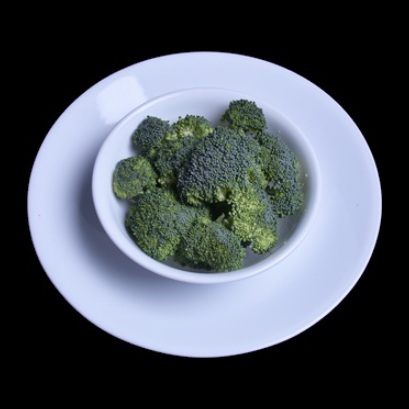

Supplement: Supplementary file 4 [file Data_Sheet_2.ZIP › broccoli.jpg]

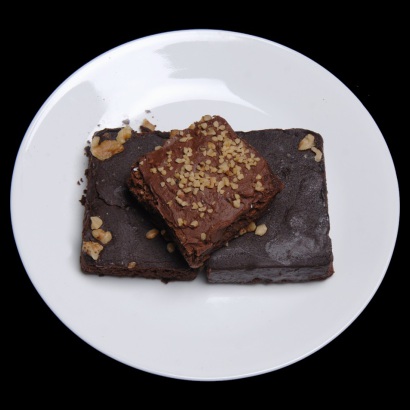

Supplement: Supplementary file 4 [file Data_Sheet_2.ZIP › brownie.jpg]

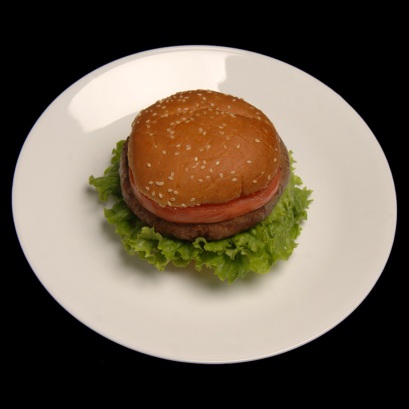

Supplement: Supplementary file 4 [file Data_Sheet_2.ZIP › burger.jpg]

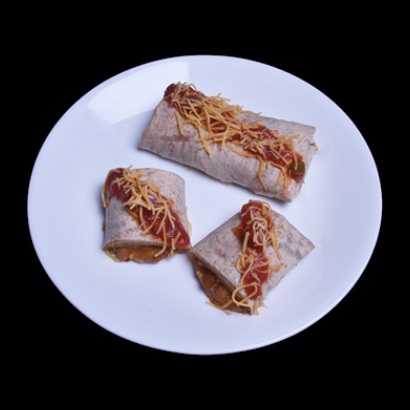

Supplement: Supplementary file 4 [file Data_Sheet_2.ZIP › burrito.jpg]

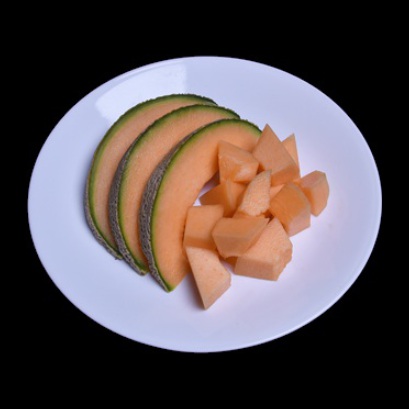

Supplement: Supplementary file 4 [file Data_Sheet_2.ZIP › cantaloupe.jpg]

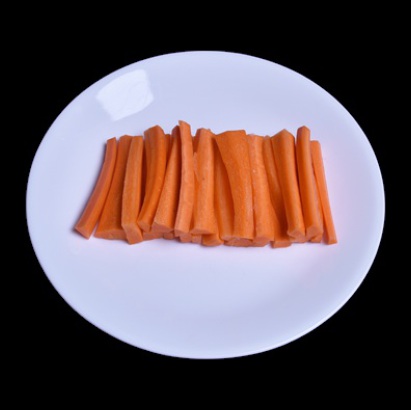

Supplement: Supplementary file 4 [file Data_Sheet_2.ZIP › carrotsticks.jpg]

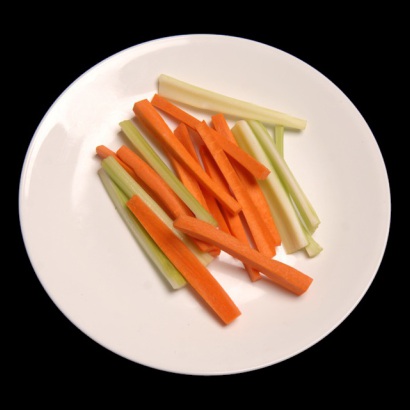

Supplement: Supplementary file 4 [file Data_Sheet_2.ZIP › celery and carrot sticks.jpg]

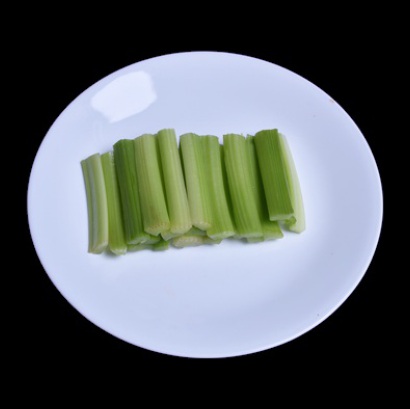

Supplement: Supplementary file 4 [file Data_Sheet_2.ZIP › celerysticks.jpg]

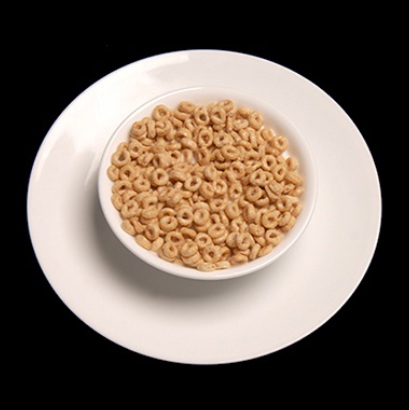

Supplement: Supplementary file 4 [file Data_Sheet_2.ZIP › cheerios.jpg]

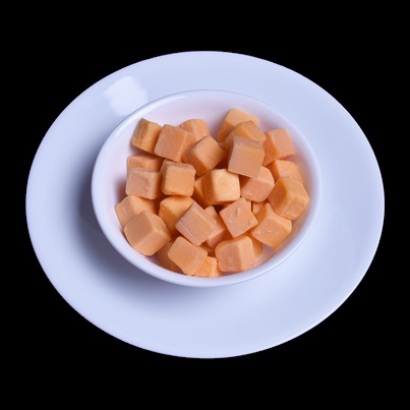

Supplement: Supplementary file 4 [file Data_Sheet_2.ZIP › cheesecubes.jpg]

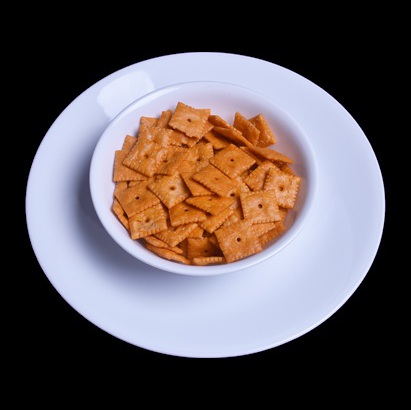

Supplement: Supplementary file 4 [file Data_Sheet_2.ZIP › cheezeits.jpg]

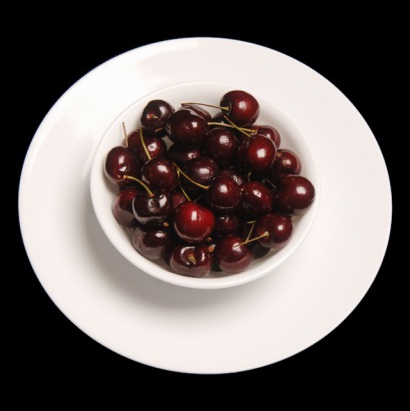

Supplement: Supplementary file 4 [file Data_Sheet_2.ZIP › cherries.jpg]

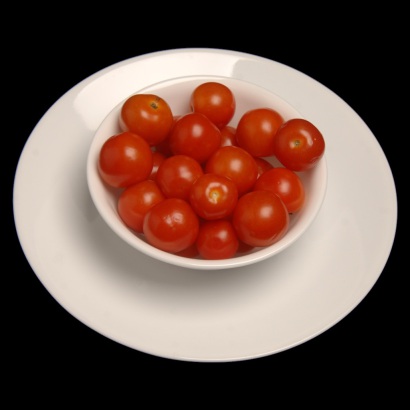

Supplement: Supplementary file 4 [file Data_Sheet_2.ZIP › cherry tomato.jpg]

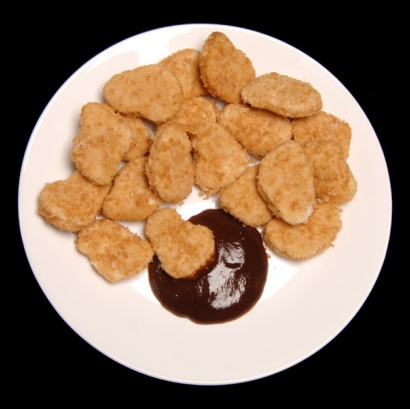

Supplement: Supplementary file 4 [file Data_Sheet_2.ZIP › chicken nuggets2.jpg]

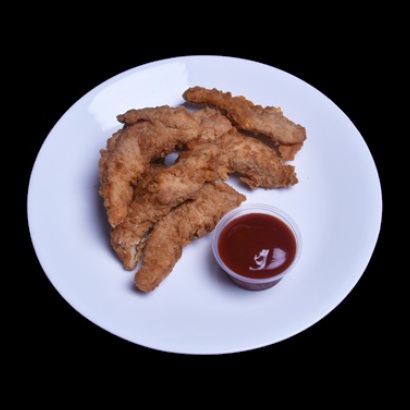

Supplement: Supplementary file 4 [file Data_Sheet_2.ZIP › chickenfingers.jpg]

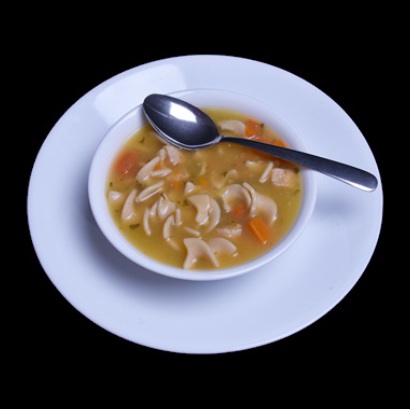

Supplement: Supplementary file 4 [file Data_Sheet_2.ZIP › chickennoodlesoup.jpg]

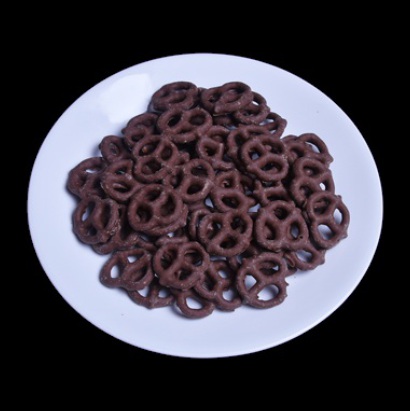

Supplement: Supplementary file 4 [file Data_Sheet_2.ZIP › chocolatecoveredpretzels.jpg]

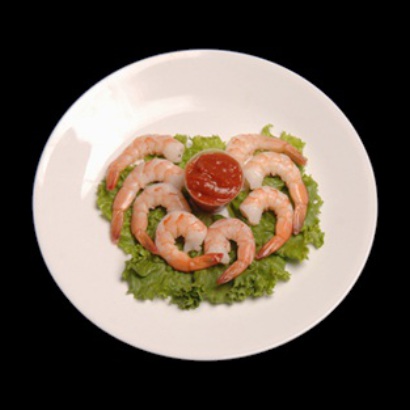

Supplement: Supplementary file 4 [file Data_Sheet_2.ZIP › cocktailshrimp.jpg]

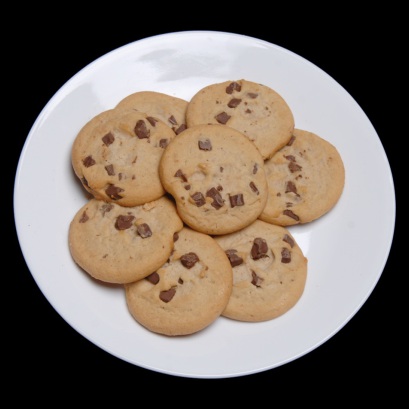

Supplement: Supplementary file 4 [file Data_Sheet_2.ZIP › cookies.jpg]

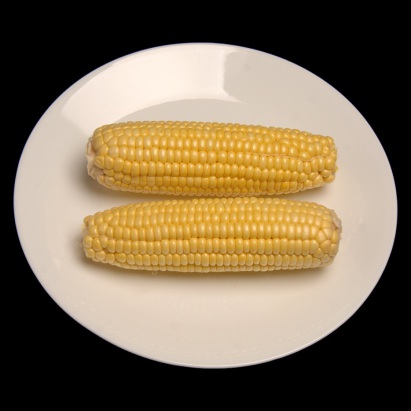

Supplement: Supplementary file 4 [file Data_Sheet_2.ZIP › corn cob.jpg]

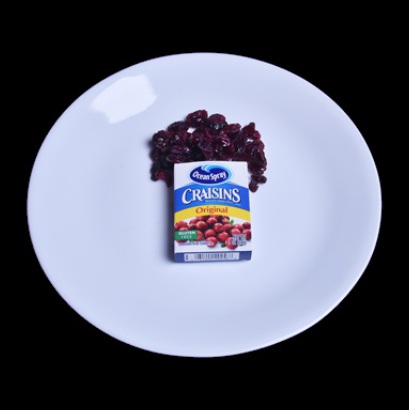

Supplement: Supplementary file 4 [file Data_Sheet_2.ZIP › craisins.jpg]

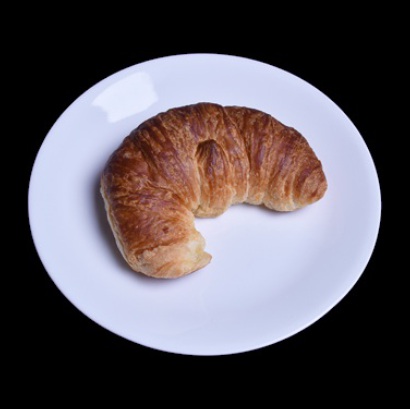

Supplement: Supplementary file 4 [file Data_Sheet_2.ZIP › croissant.jpg]

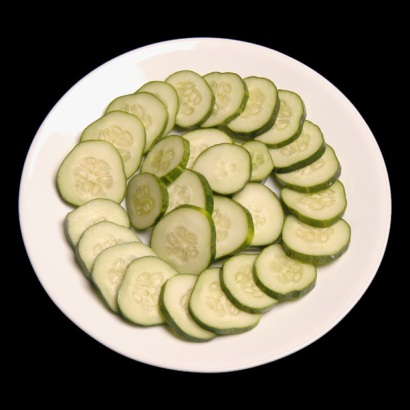

Supplement: Supplementary file 4 [file Data_Sheet_2.ZIP › cucumber slices.jpg]

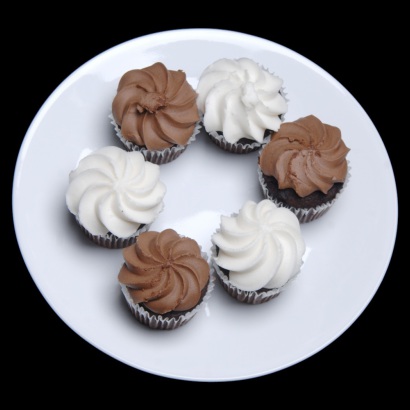

Supplement: Supplementary file 4 [file Data_Sheet_2.ZIP › cupcakes am.jpg]

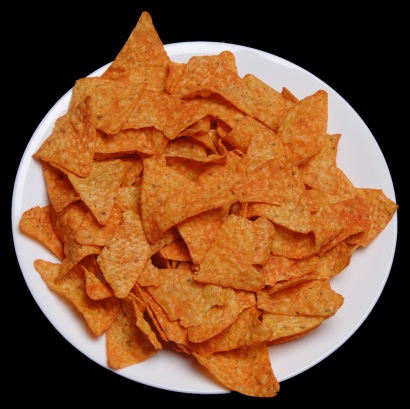

Supplement: Supplementary file 4 [file Data_Sheet_2.ZIP › doritos.jpg]

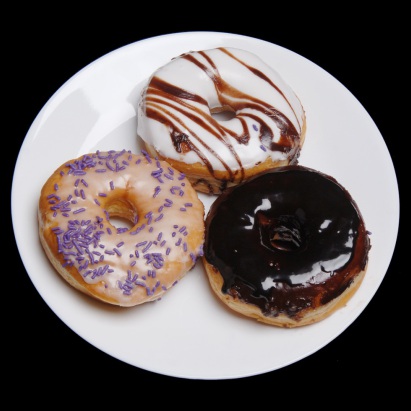

Supplement: Supplementary file 4 [file Data_Sheet_2.ZIP › doughnuts.jpg]

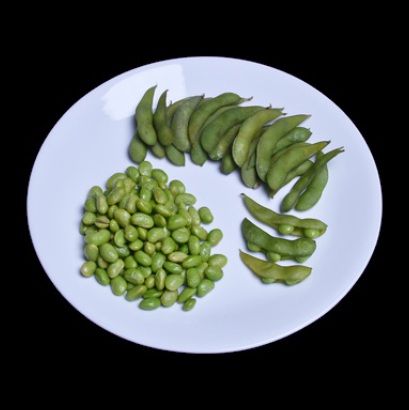

Supplement: Supplementary file 4 [file Data_Sheet_2.ZIP › edamame.jpg]

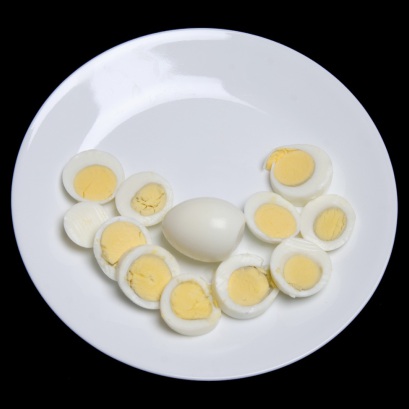

Supplement: Supplementary file 4 [file Data_Sheet_2.ZIP › eggs.jpg]

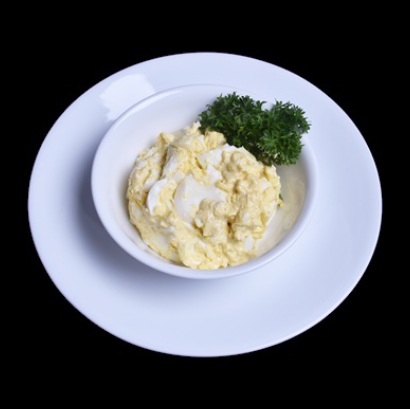

Supplement: Supplementary file 4 [file Data_Sheet_2.ZIP › eggsalad.jpg]

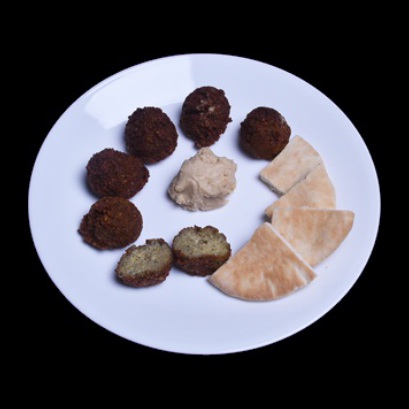

Supplement: Supplementary file 4 [file Data_Sheet_2.ZIP › falafelwithpita.jpg]

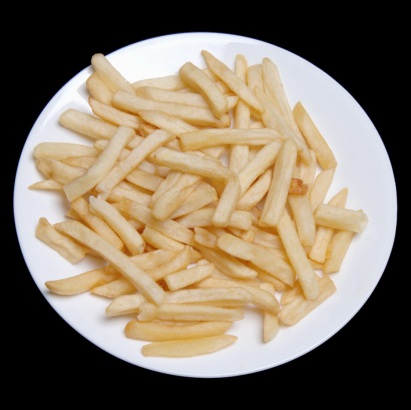

Supplement: Supplementary file 4 [file Data_Sheet_2.ZIP › french fries.jpg]

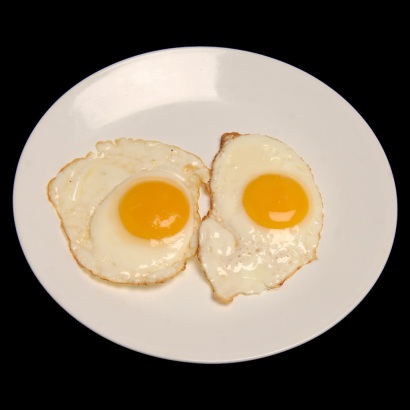

Supplement: Supplementary file 4 [file Data_Sheet_2.ZIP › fried eggs.jpg]

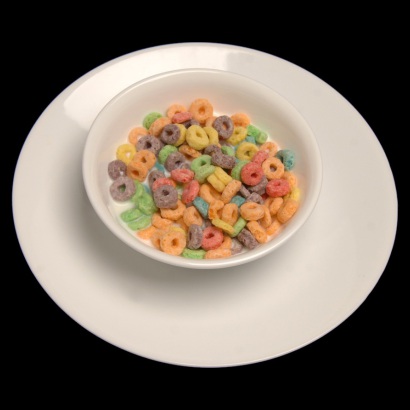

Supplement: Supplementary file 4 [file Data_Sheet_2.ZIP › froot loops w milk.jpg]

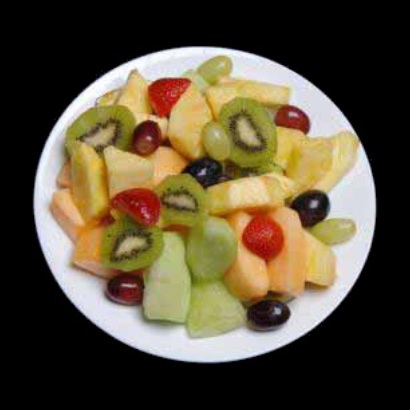

Supplement: Supplementary file 4 [file Data_Sheet_2.ZIP › fruitsalad.jpg]

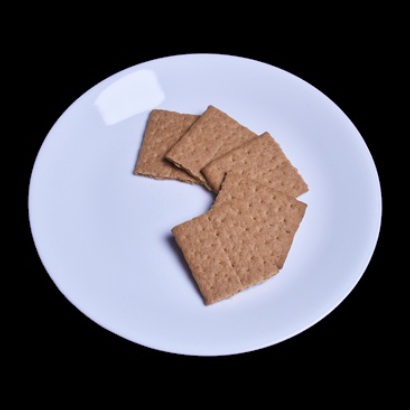

Supplement: Supplementary file 4 [file Data_Sheet_2.ZIP › grahamcrackers.jpg]

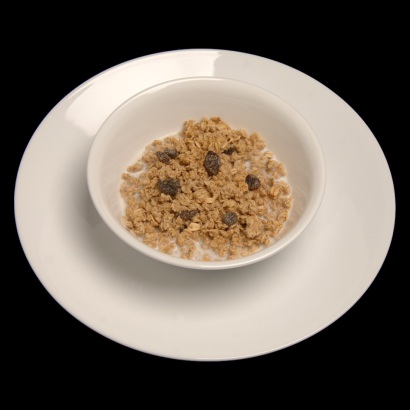

Supplement: Supplementary file 4 [file Data_Sheet_2.ZIP › granola w milk.jpg]

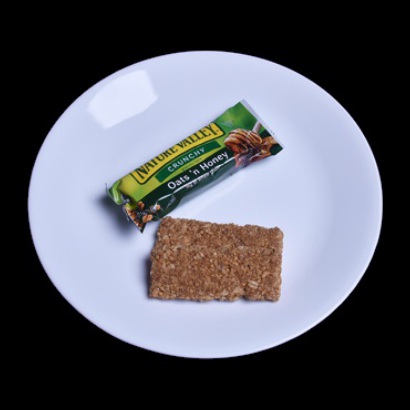

Supplement: Supplementary file 4 [file Data_Sheet_2.ZIP › granolabar.jpg]

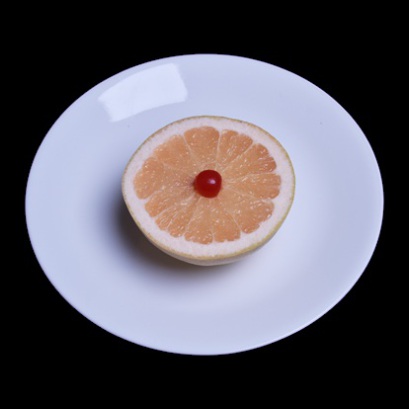

Supplement: Supplementary file 4 [file Data_Sheet_2.ZIP › grapefruit.jpg]

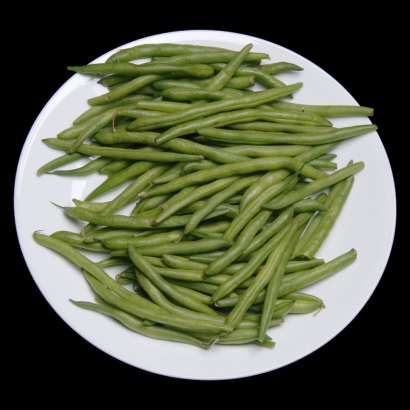

Supplement: Supplementary file 4 [file Data_Sheet_2.ZIP › green beans.jpg]

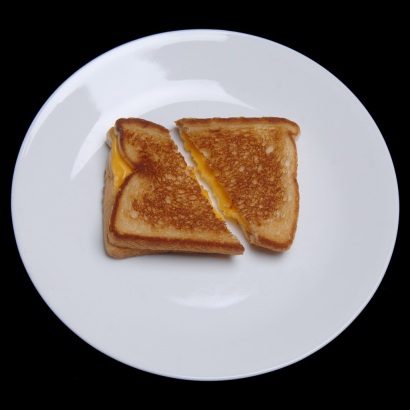

Supplement: Supplementary file 4 [file Data_Sheet_2.ZIP › grilled cheese.jpg]

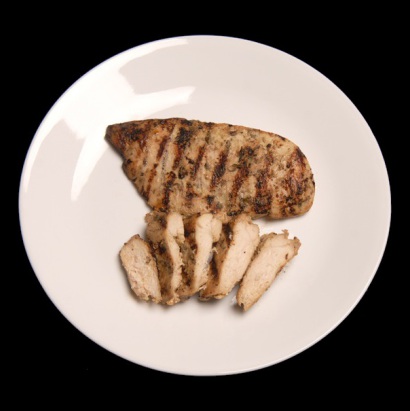

Supplement: Supplementary file 4 [file Data_Sheet_2.ZIP › grilled chicken strips.jpg]

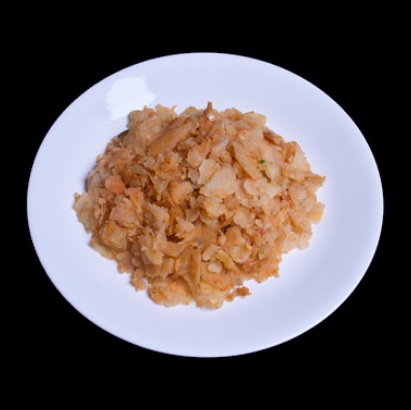

Supplement: Supplementary file 4 [file Data_Sheet_2.ZIP › hashbrowns.jpg]

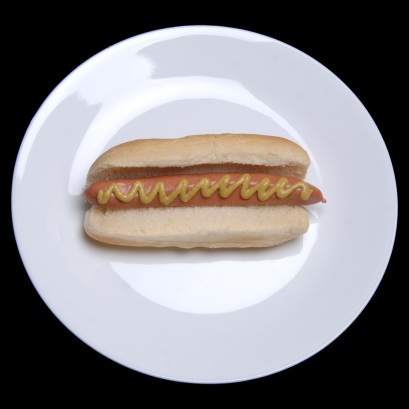

Supplement: Supplementary file 4 [file Data_Sheet_2.ZIP › hotdog w must.jpg]

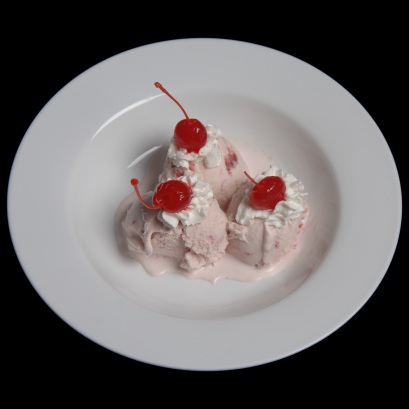

Supplement: Supplementary file 4 [file Data_Sheet_2.ZIP › ice cream sundae 002.jpg]

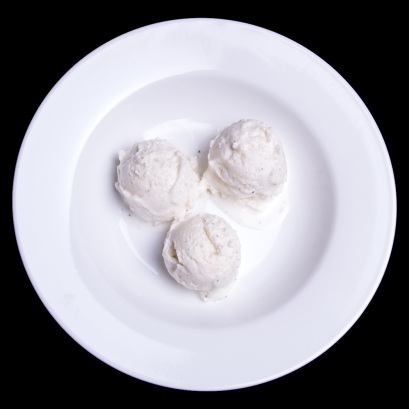

Supplement: Supplementary file 4 [file Data_Sheet_2.ZIP › icecream.jpg]

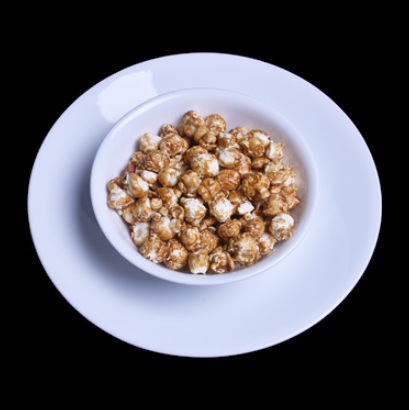

Supplement: Supplementary file 4 [file Data_Sheet_2.ZIP › kettlecorn.jpg]

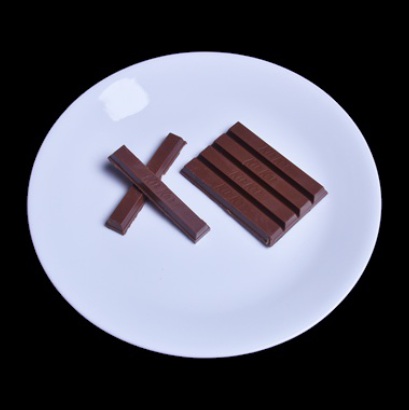

Supplement: Supplementary file 4 [file Data_Sheet_2.ZIP › kitkat.jpg]

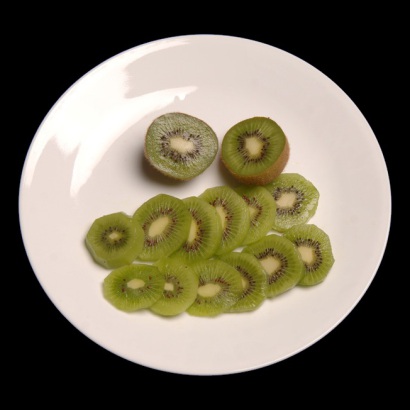

Supplement: Supplementary file 4 [file Data_Sheet_2.ZIP › kiwi.jpg]

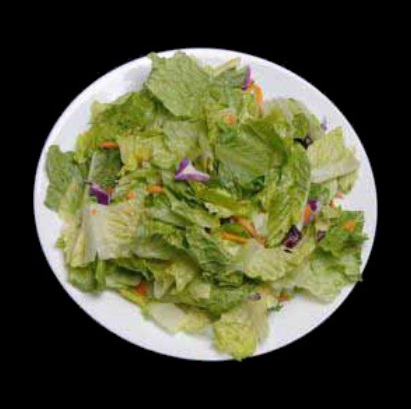

Supplement: Supplementary file 4 [file Data_Sheet_2.ZIP › lettucesalad.jpg]

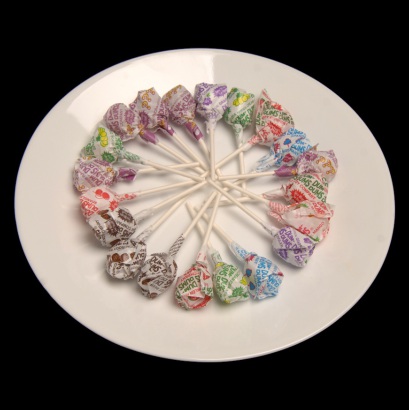

Supplement: Supplementary file 4 [file Data_Sheet_2.ZIP › lollipops.jpg]

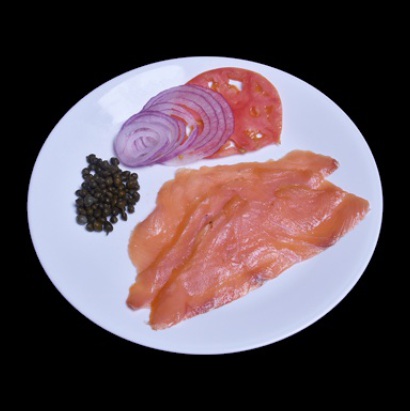

Supplement: Supplementary file 4 [file Data_Sheet_2.ZIP › lox.jpg]

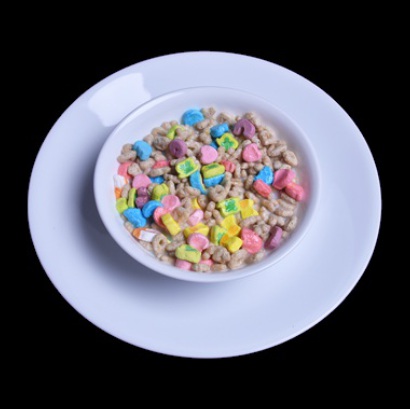

Supplement: Supplementary file 4 [file Data_Sheet_2.ZIP › luckycharms.jpg]

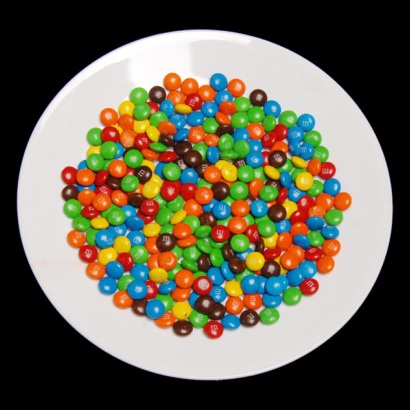

Supplement: Supplementary file 4 [file Data_Sheet_2.ZIP › m_and_m.jpg]

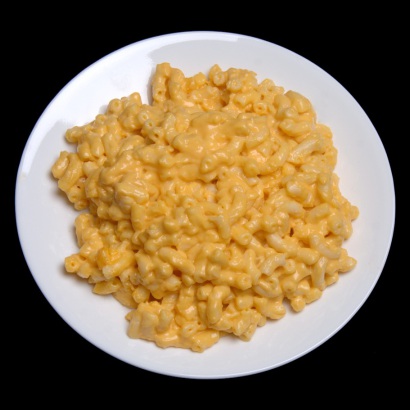

Supplement: Supplementary file 4 [file Data_Sheet_2.ZIP › mac & cheese.jpg]

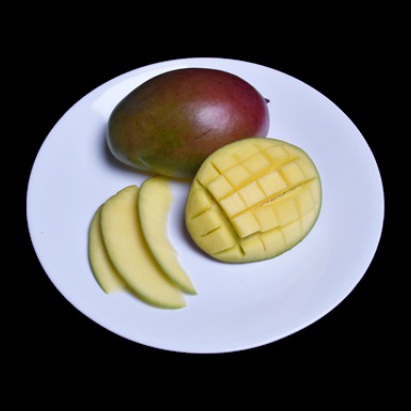

Supplement: Supplementary file 4 [file Data_Sheet_2.ZIP › mango.jpg]

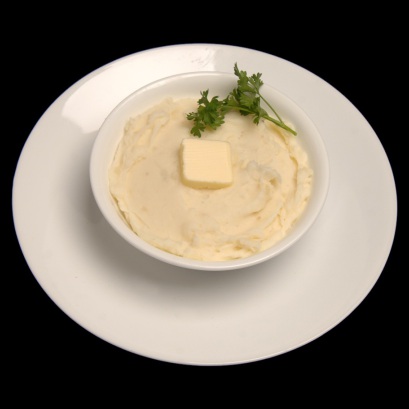

Supplement: Supplementary file 4 [file Data_Sheet_2.ZIP › mashed potato.jpg]

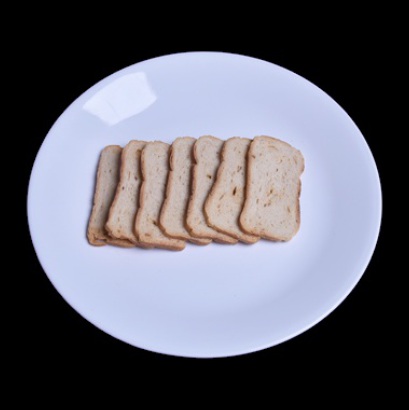

Supplement: Supplementary file 4 [file Data_Sheet_2.ZIP › melbatoastcrackers.jpg]

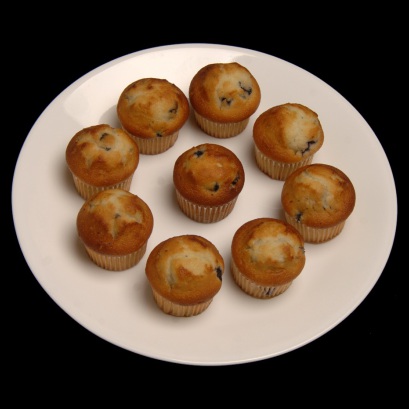

Supplement: Supplementary file 4 [file Data_Sheet_2.ZIP › mini muffins.jpg]

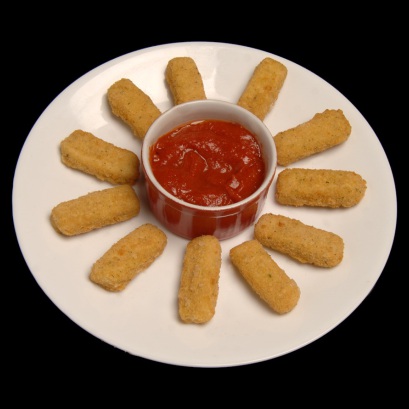

Supplement: Supplementary file 4 [file Data_Sheet_2.ZIP › mozarella sticks.jpg]

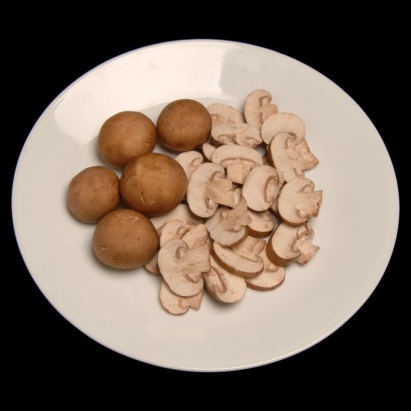

Supplement: Supplementary file 4 [file Data_Sheet_2.ZIP › mushrooms.jpg]

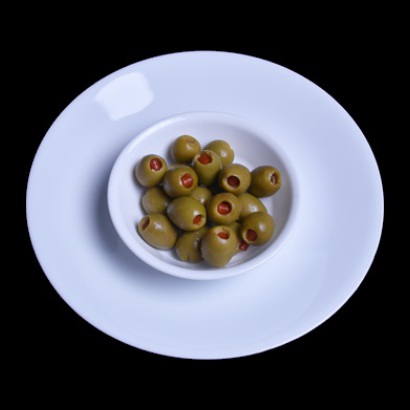

Supplement: Supplementary file 4 [file Data_Sheet_2.ZIP › olives.jpg]

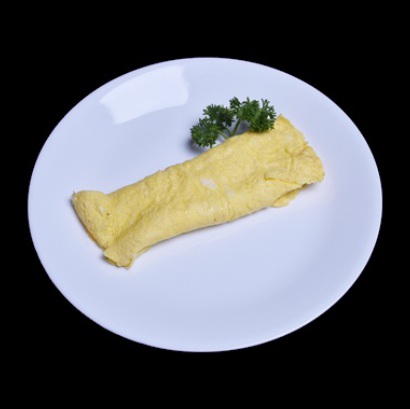

Supplement: Supplementary file 4 [file Data_Sheet_2.ZIP › omelette.jpg]

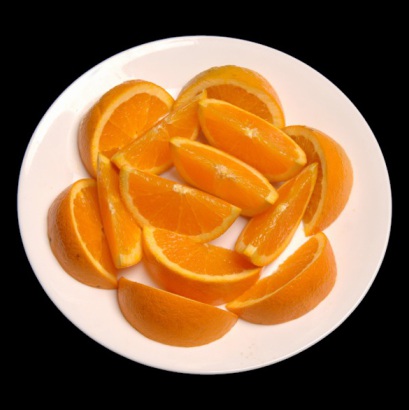

Supplement: Supplementary file 4 [file Data_Sheet_2.ZIP › orange slices.jpg]

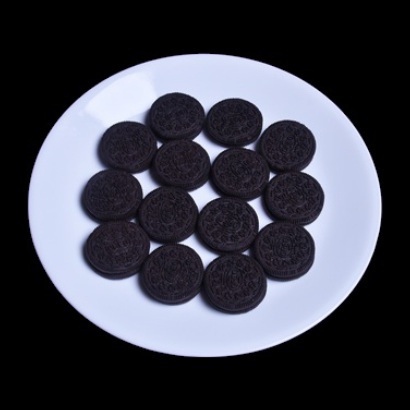

Supplement: Supplementary file 4 [file Data_Sheet_2.ZIP › oreos.jpg]

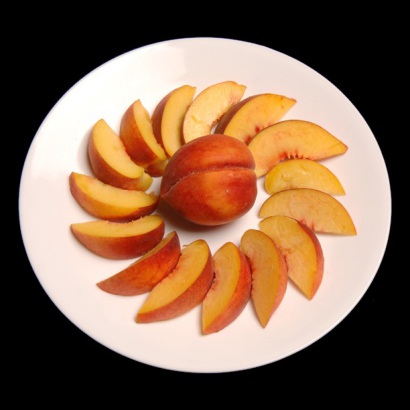

Supplement: Supplementary file 4 [file Data_Sheet_2.ZIP › peaches.jpg]

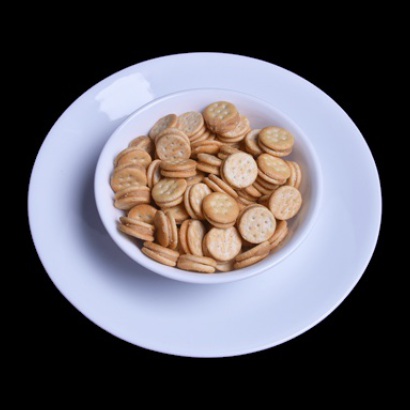

Supplement: Supplementary file 4 [file Data_Sheet_2.ZIP › peanutbutterritz.jpg]

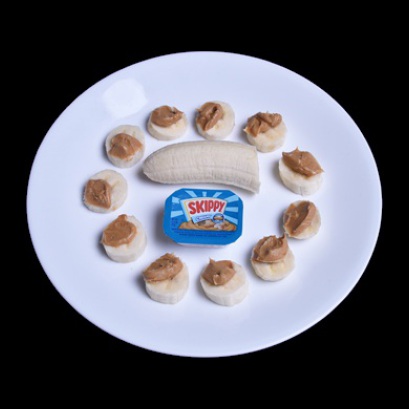

Supplement: Supplementary file 4 [file Data_Sheet_2.ZIP › peanutbutterwithbanana.jpg]

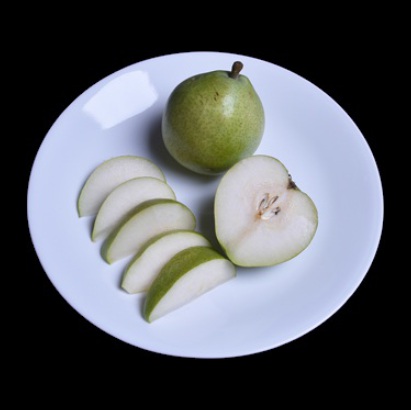

Supplement: Supplementary file 4 [file Data_Sheet_2.ZIP › pear.jpg]

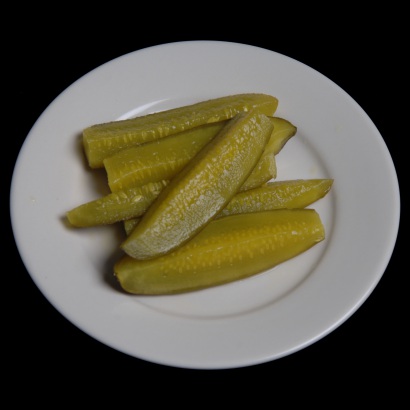

Supplement: Supplementary file 4 [file Data_Sheet_2.ZIP › pickles.jpg]

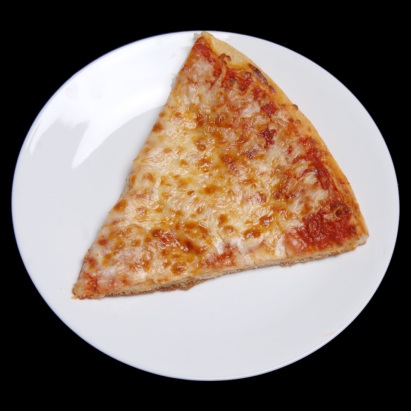

Supplement: Supplementary file 4 [file Data_Sheet_2.ZIP › pizza.jpg]

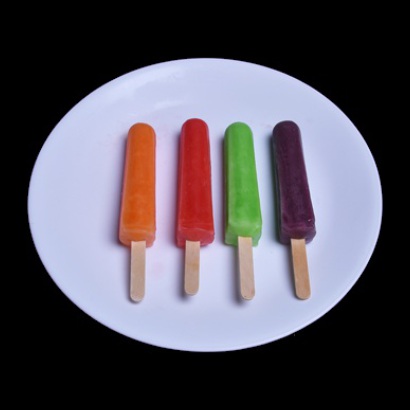

Supplement: Supplementary file 4 [file Data_Sheet_2.ZIP › popsicles.jpg]

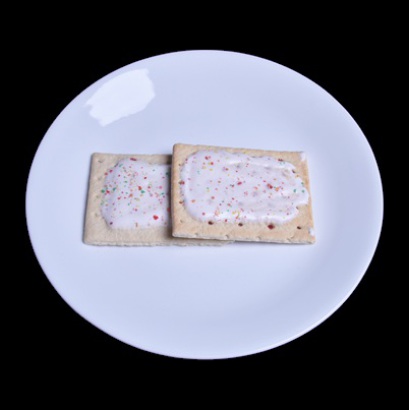

Supplement: Supplementary file 4 [file Data_Sheet_2.ZIP › poptarts.jpg]

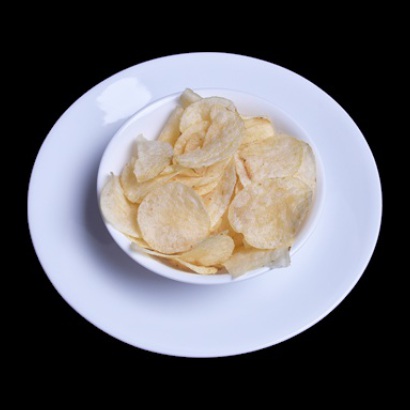

Supplement: Supplementary file 4 [file Data_Sheet_2.ZIP › potatochips.jpg]

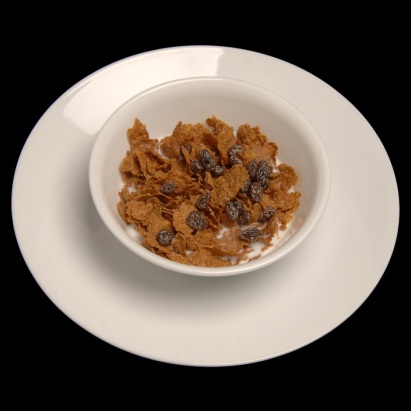

Supplement: Supplementary file 4 [file Data_Sheet_2.ZIP › raisin bran w milk.jpg]

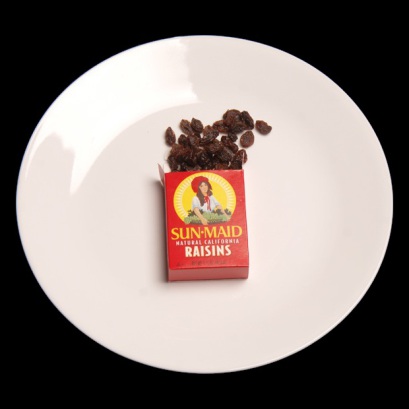

Supplement: Supplementary file 4 [file Data_Sheet_2.ZIP › raisins.jpg]

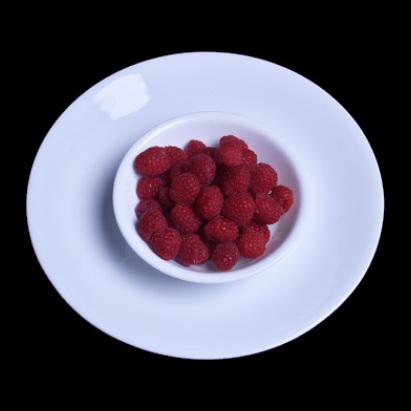

Supplement: Supplementary file 4 [file Data_Sheet_2.ZIP › raspberries.jpg]

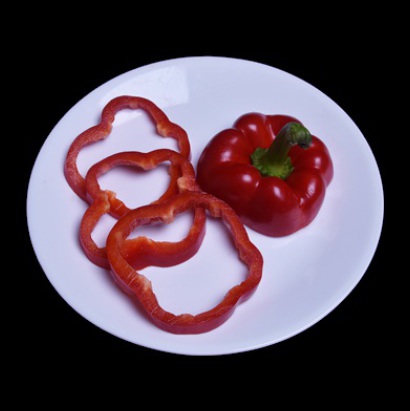

Supplement: Supplementary file 4 [file Data_Sheet_2.ZIP › redbellpepper.jpg]

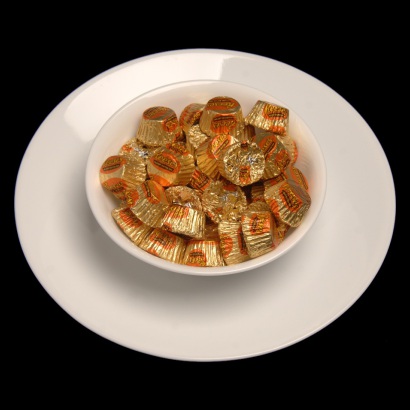

Supplement: Supplementary file 4 [file Data_Sheet_2.ZIP › reeses.jpg]
